# Evaluation of Antarctic Ozone Profiles derived from OMPS-LP by using Balloon-borne Ozonesondes

**DOI:** 10.1038/s41598-021-81954-6

**Published:** 2021-02-22

**Authors:** Edgardo Sepúlveda, Raul R. Cordero, Alessandro Damiani, Sarah Feron, Jaime Pizarro, Felix Zamorano, Rigel Kivi, Ricardo Sánchez, Margarita Yela, Julien Jumelet, Alejandro Godoy, Jorge Carrasco, Juan S. Crespo, Gunther Seckmeyer, Jose A. Jorquera, Juan M. Carrera, Braulio Valdevenito, Sergio Cabrera, Alberto Redondas, Penny M. Rowe

**Affiliations:** 1 Universidad de Santiago de Chile, Av. B. O’Higgins 3363, Santiago, Chile; 2 Center of Environmental Remote Sensing, Chiba University, Chiba, Japan; 3 Department of Earth System Science, Stanford University, Stanford, CA 94305-2210 USA; 4 University of Magallanes, Punta Arenas, Chile; 5 Space and Earth Observation Centre, Finnish Meteorological Institute (FMI), Sodankylä, Finland; 6 Servicio Meteorológico Nacional, Buenos Aires, Argentina; 7 Instituto Nacional de Técnica Aeroespacial (INTA), Madrid, Spain; 8 LATMOS/IPSL, Sorbonne Université, UVSQ, CNRS, Paris, France; 9 Dirección Meteorológica de Chile, Santiago, Chile; 10 Leibniz Universität Hannover, Herrenhauser Strasse 2, Hannover, Germany; 11 Instituto de Ciencias Biomédicas, Universidad de Chile, Santiago, Chile; 12 Izaña Atmospheric Research Center (IARC), State Meteorological Agency (AEMET), Santa Cruz de Tenerife, Spain; 13 NorthWest Research Associates, Redmond, WA USA

**Keywords:** Climate sciences, Atmospheric science

## Abstract

Predicting radiative forcing due to Antarctic stratospheric ozone recovery requires detecting changes in the ozone vertical distribution. In this endeavor, the Limb Profiler of the Ozone Mapping and Profiler Suite (OMPS-LP), aboard the Suomi NPP satellite, has played a key role providing ozone profiles over Antarctica since 2011. Here, we compare ozone profiles derived from OMPS-LP data (version 2.5 algorithm) with balloon-borne ozonesondes launched from 8 Antarctic stations over the period 2012–2020. Comparisons focus on the layer from 12.5 to 27.5 km and include ozone profiles retrieved during the Sudden Stratospheric Warming (SSW) event registered in Spring 2019. We found that, over the period December-January–February-March, the root mean square error (*RMSE*) tends to be larger (about 20%) in the lower stratosphere (12.5–17.5 km) and smaller (about 10%) within higher layers (17.5–27.5 km). During the ozone hole season (September–October–November), *RMSE* values rise up to 40% within the layer from 12.5 to 22 km. Nevertheless, relative to balloon-borne measurements, the mean bias error of OMPS-derived Antarctic ozone profiles is generally lower than 0.3 ppmv, regardless of the season.

## Introduction

The stratospheric ozone layer protects life on Earth by absorbing energetic and harmful ultraviolet (UV) radiation^1–6^. Ozone depletion due to human-made ozone depleting substances (ODSs) acquired global resonance after the discovery of the Antarctic ozone hole^7^. The ozone hole is a seasonal phenomenon of strong ozone depletion, which occurs in Antarctica every year. A strong stratospheric jet stream (i.e., the polar night jet) develops along the boundary of sunlight and polar winter darkness and, by confining air within the polar vortex, favors low stratospheric temperatures needed for the formation of Polar Stratospheric Clouds (PSCs). The PSCs provide a reaction site for heterogeneous chemical reactions involving the ODSs, which leads to the catalytic ozone destruction when sunlight returns to Antarctica^8^. As a consequence, the ozone within the layer from 15 to 20 km is almost totally depleted throughout the early spring^9^ until temperatures warm and the polar vortex weakens, ending the isolation of the air in the polar vortex.

Responding to the ozone depletion, the Montreal Protocol banned numerous human-made ODSs^10^. Nearly 30 years after the Montreal Protocol came into effect, satellite-derived data show that Antarctic ODS levels are declining^10,11^ and that the Antarctic ozone abundance has begun to increase^12,13^. Although the evolution of stratospheric ozone will be determined not only by the decline in ODSs but also by the increase in greenhouse gases (GHGs), Antarctic stratospheric ozone is expected to recover back to the 1980 level in the 2060s^10^.

Ozone also plays a role in the radiative budget, affecting the atmospheric circulation and climate. Even small variations in the distribution of trace gases, like ozone, can significantly impact the radiative forcing of the Earth’s climate and are of key importance for understanding climate change^14^. Recent atmospheric circulation changes in the southern hemisphere have been attributed to rising greenhouse-gas concentrations and Antarctic ozone depletion^15–19^. The acceleration and deceleration of the Brewer-Dobson circulation (BDC) have also been linked with changes in the ozone abundance over Antarctica^20,21^. Therefore, a better understanding of the transport related processes that control the concentrations of radiatively and chemically active species like ozone is of great interest.

Predicting the radiative forcing due to stratospheric ozone recovery and related processes during this century also requires detecting changes in the vertical distribution of ozone^22^. In this endeavor, satellite-derived estimates play a key role, especially when ground based measurements are scarce. Numerous efforts have focused on the validation of satellite estimates of the total ozone column^23–30^. Less attention has been paid to ozone profiles, such as those produced by the Ozone Mapping and Profiler Suite (OMPS), aboard the Suomi National Polar-orbiting Partnership (Suomi NPP) satellite, orbiting since October 2011. As part of the Joint Polar Satellite System (JPSS)^31^ of the National Aeronautics and Space Administration (NASA) and the National Oceanic and Atmospheric Administration (NOAA), the OMPS provides estimates of both total ozone columns and ozone profiles^31,32^.

A method used to evaluate limb profilers is based on the comparison with balloon-borne ozonesondes^33^. Here, we present a comparison between “last state” OMPS-LP ozone profiles (version 2.5 algorithm) and balloon-borne measurements of the vertical distribution of ozone based on the Electrochemical Concentration Cell (ECC) method. Balloon-borne ozonesondes were launched from 8 Antarctic stations over the period 2012–2020. Our comparisons focus on the layer from 12.5 to 27 km (within which satellite-derives profiles and balloon-borne data overlap) and include ozone profiles retrieved during the Sudden Stratospheric Warming (SSW) event registered in Spring 2019^34–37^. Comparisons consider two periods: September–October–November (SON), when the Antarctic ozone hole occurs, and December-January–February-March (DJFM), when the ozone abundance returns to nearly normal values. OMPS-LP data are not available over Antarctica from April to August.

## Methods

### Satellite-derived estimates

The OMPS is a suite of three detectors measuring solar radiances scattered by the atmosphere and solar irradiance in overlapping spectral ranges. These sensors are the OMPS Nadir Mapper (OMPS-NM) for total ozone column measurements, the OMPS Nadir Profiler (OMPS-NP) for low vertical resolution ozone profiles (12 Umkehr Layers), and the OMPS Limb Profiler (OMPS-LP) for high vertical resolution ozone profiles with a vertical range of 12.5–60 km.

The OMPS-LP instrument is a limb sensor type, which observes the “edge” of the Earth´s atmosphere (or earth’s limb). The closest approach of the sensor line of sight to the Earth´s surface is referred to as the tangent point; this is the point where the sensor line of sight intersects an Earth radius vector at a right angle and where the retrieval algorithms calculate the ozone amounts^38^. The limb view tangent points pass each geographic location approximately 7 min after they are viewed by the nadir instrument. The OMPS-LP produces 160–180 limb measurements per orbit, with 14–15 orbits per day^39,40^, and 19 s as the record frequency (125 km along-track motion). The limb measurements consist of the detection of scattered radiances in the line of sight, which then lead to ozone abundances through the use of an iterative procedure that involves a radiative transfer model^41–44^. The OMPS-LP Charge Coupled Device (CCD) detector provides measurements every 1.1 km with 2.1 km vertical resolution. The expected precision for ozone retrievals is better than 20% (or 0.1 ppmv) for elevations lower than 25 km and 5–10% for elevations from 25–50 km^38^.

Three algorithm versions for ozone LP retrievals have been proposed since 2012, with the first version released just after the beginning of operations, a second version in 2014, and version 2.5, released in 2017^39^. Data from the latter version were obtained from the NASA Goddard Space Flight Center web site (Aura Validation Data Center)^32,45^.

### Balloon-borne data

**Figure 1:**
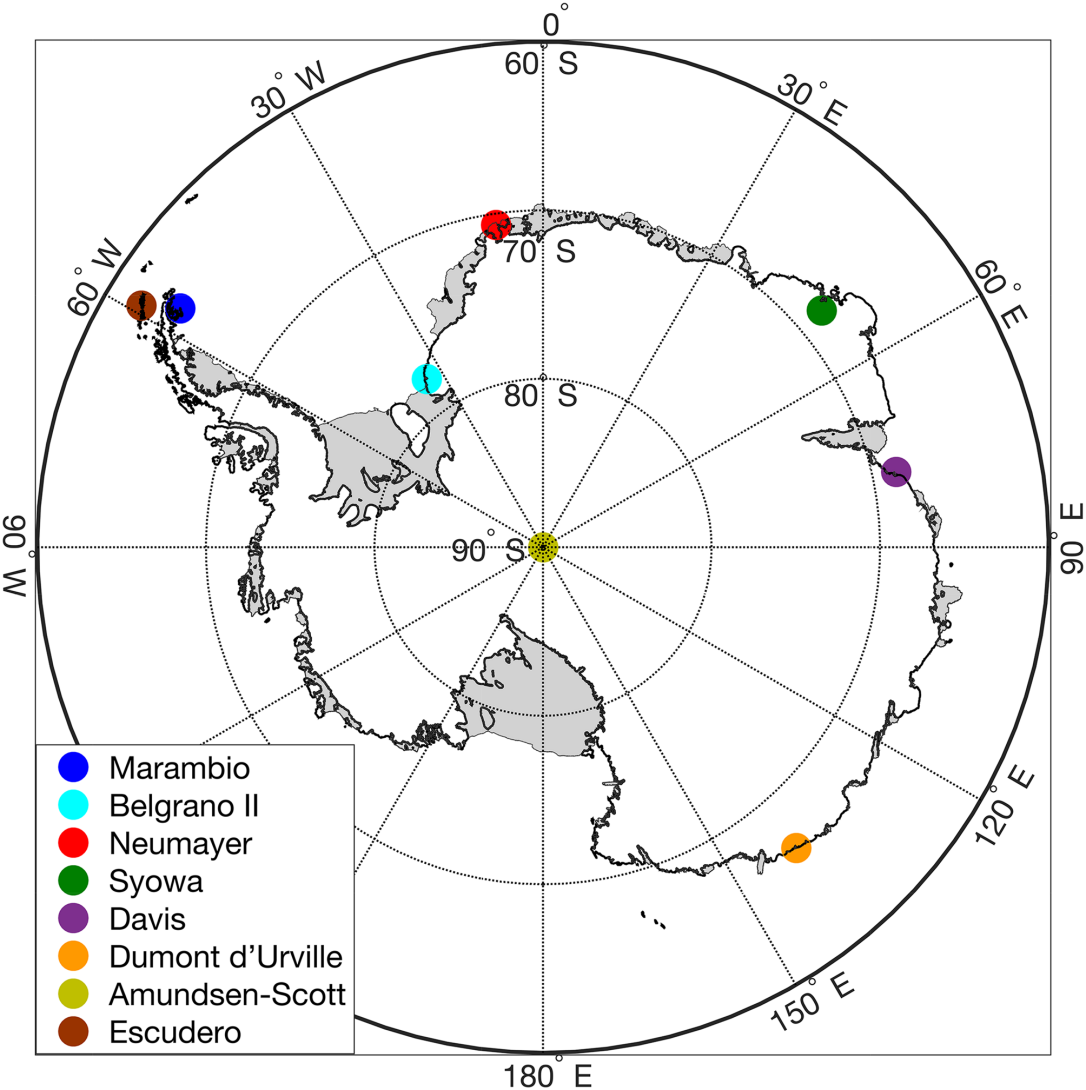
Balloon-borne ozonesonde launch sites. Map was generated by using Python’s Matplotlib Library (version 3.3.3; https://matplotlib.org/users/installing.html)^63^.

Ozone profiles can also be measured from Electrochemical Concentration Cell (ECC) ozonesondes. Balloon-borne ozonesondes have been regularly launched from eight Antarctic stations (see Fig. 1 and Table 1), over the period 2012–2019. These data are available from the World Ozone and UV Data Center (WOUDC) and the Network for the Detection of Atmospheric Composition Change (NDACC). Additional ozone profiles were obtained from a campaign at Escudero Station on King George Island (62.2^o^S, 58.9^o^W) conducted in spring 2019 in the frame of the SouthTRAC-Halo project and during a SSW event registered in Spring 2019^34–37^. During that special campaign period, 16 ECC ozonesondes, connected to GRAW DFM-09 radiosondes were launched.

**Table 1:** Number of ozone profiles considered in the comparisons, over the periods: DJFM 2013–2020, SON 2012–2018, and SON 2019.

Station	Latitude	Longitude	Data Network	Period	Profiles	DJFM 2013–2020	SON 2012–2018	SON 2019
Marambio	− 64.24	− 56.63	WOUDC	2012–2020	128	47	81	0
Belgrano II	− 77.87	− 34.63	NDACC	2016–2020	19	0	15	4
Neumayer	− 70.67	− 8.27	NDACC	2012–2020	312	114	179	19
Syowa	− 68.30	49.64	WOUDC	2012–2020	148	52	83	13
Davis	− 68.57	77.97	WOUDC	2012–2020	94	34	60	0
Dumont d’Urville	− 66.66	139.91	NDACC	2012–2020	53	16	37	0
Amundsen-Scott	− 89.98	139.28	NDACC	2012–2020	129	74	49	6
Escudero	− 62.20	− 58.92	–	2019	5	0	0	5

Balloon-borne ozonesondes (attached to a radiosonde) allow for measurements of the vertical distribution of ozone concentration, temperature, relative humidity (RH), pressure, and winds up to about 30 km. This system is considered to be the most accurate manner to measure high vertical resolution profiles^46^. The ECC ozonesondes systems are mainly composed of a battery-powered gas-sampling pump and an ozone sensor made of two electrodes immersed in potassium iodide (KI) solutions of different concentrations, contained in separate cathode and anode chambers. The measurement is based on the titration of ozone in the KI sensing solution, producing an electrical signal from the difference in concentration of the KI-solution between the chambers^47,48^. The detection limit of ECC ozonesondes is typically less than 2 ppbv, while the associated uncertainty is about 10% in the troposphere and 5% in the stratosphere up to 10 hPa, and 5–25% between 10 and 3 hPa^49–55^.

### Comparison criteria

Following prior efforts^32,40^, we adopted spatial and temporal requirements for the OMPS-LP and ozonesonde comparison studies. Satellite profiles should be less than 500 km from the ozonesonde launch site, and within a time span of ± 12 h. For Amundsen-Scott Station’s sondes, the required distance was increased to 1000 km due to the orbit track of the OMPS-LP (the average distance is 960 km in the case of the South Pole^40^). Comparisons focus on the layer from 12.5 to 27.5 km (within which satellite-derived and balloon-borne data overlap). Since OMPS-LP data from mid-April to late August are not available over Antarctica, comparisons were conducted over two periods: September–October–November (SON) when the Antarctic ozone hole occurs, and December-January–February–March (DJFM) when the ozone abundance returns to nearly normal values. In addition, we also compared ozone profiles retrieved during the SSW event registered over the period SON 2019^34–37^. Before the comparisons, ozonesonde profiles were interpolated to a common 5-m vertical grid and then convolved with a Gaussian averaging kernel over a 1 km range around each sonde grid point.

For each station and at each altitude, we computed the mean bias error (*MBE*) and the root mean square error (*RMSE*) of OMPS-LP-derived estimates of ozone relative to balloon-borne data; the correlation coefficient (*R*) at each altitude was also calculated. Moreover, we computed at each altitude the correlation between some OMPS-LP parameters and the relative differences (between the OMPS-LP-derived and balloon-borne data of ozone); the following OMPS-LP parameters were considered: time difference between satellite readings and ozonesonde launch, distance to the station, solar zenith angle (SZA), single scattering angle (SSA), tropopause altitude, surface reflectance, and cloud height. Finally, we built up scatter plots formed by clustering, regardless of altitude, OMPS-LP-derived and balloon-borne data of ozone. The results for each station are shown in the Supplementary Material.

Balloon-borne data are reported in partial pressure units (mPa), while OMPS-LP ozone profiles retrievals are presented in number density units (number of ozone molecules per cubic centimeter). The transformation from partial pressure units into number density units is straightforward if temperature profiles are available. Therefore, we began our analyses by checking the consistency of balloon-borne measurements of temperature using Modern-Era Retrospective analysis for Research and Applications, Version 2 (MERRA-2)^56^. MERRA-2 data are embedded in the OMPS-LP dataset. Reliable temperature profiles are needed for the transformation from partial pressure units into number density units of the sonde readings. At each altitude, we computed *MBE*, *RMSE* and *R* between balloon-borne measurements of temperature and MERRA-2 data^56^. As shown in Fig. S1, the differences between balloon-borne measurements of the temperature and reanalysis data are generally lower than 0.5%. The *MBE* was greater (but still less than 1%) in the case of Amundsen-Scott Station’s sondes, which can be attributed to the distance between the orbit track and the launch site.

## Results

**Figure 2:**
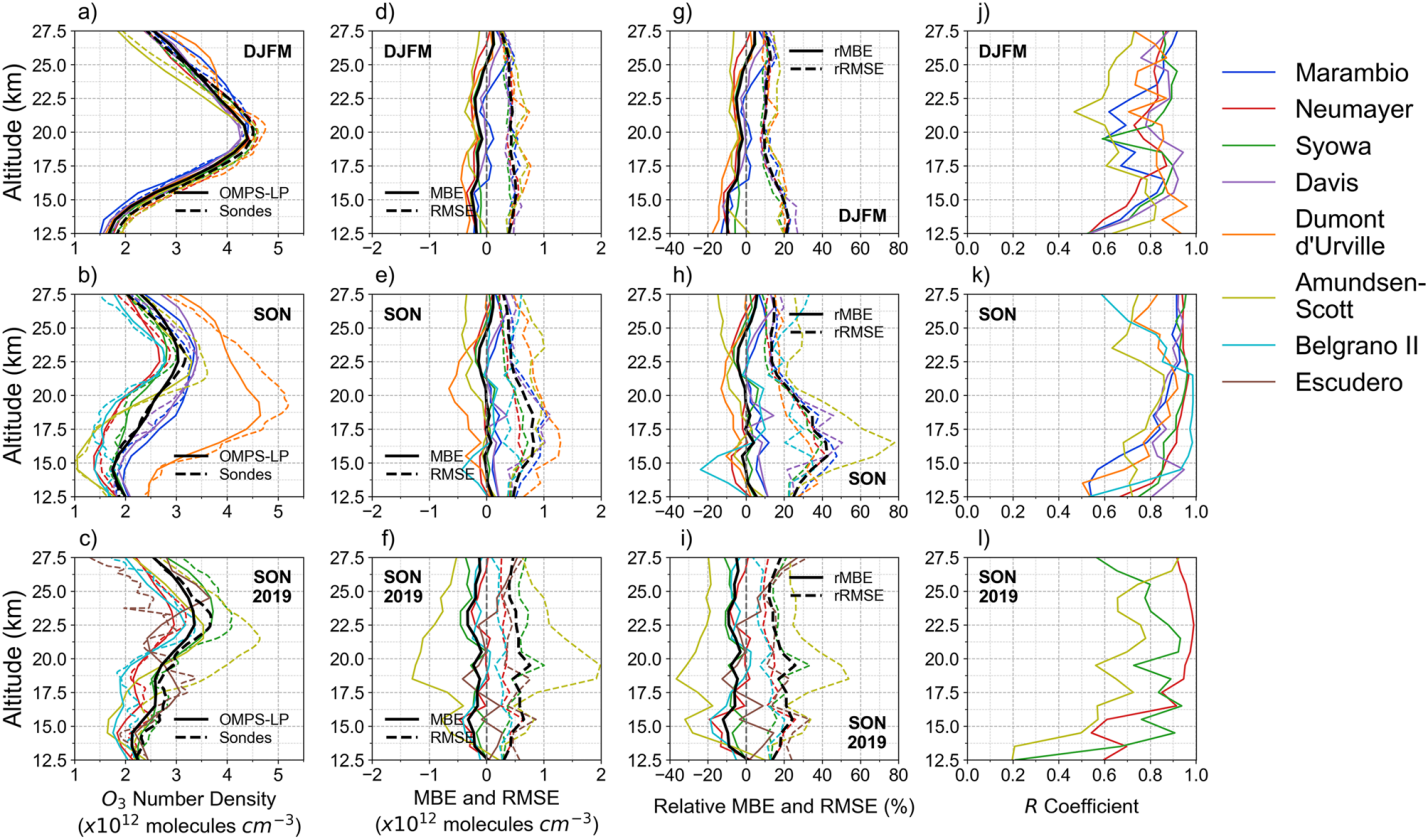
Comparison between OMPS-LP-derived and balloon-borne data of ozone over the period DJFM 2013–2020 (first row), over the period SON 2012–2018 (second row), and over the period SON 2019 (third row). (**a**–**c**) Mean profiles per station. The solid line stands for the mean computed from OMPS-LP-derived estimates of ozone and the dashed line stands for the mean computed from balloon-borne measurements of ozone. The mean was computed from the cluster formed with profiles from Marambio, Neumayer, Syowa, Davis and Dumont d’Urville stations. Amundsen-Scott Station data were not considered for computing the mean due to the long distance between the station and the orbit track; Belgrano II and Escudero data were not considered either due to the low temporal distribution of the balloon-borne ozonesondes. (**d**–**f**) Mean Bias Error (*MBE*, solid line) and Root Mean Square Error (*RMSE*, dashed line) relative to balloon-borne data. (**g**–**i**) Relative Mean Bias Error (*rMBE*, solid line) and Relative Root Mean Square Error (*rRMSE*, dashed line). (**j**–**l**) Correlation coefficient (*R*) profile; data corresponding to Belgrano II and Escudero were not considered since few balloon-borne ozonesondes during SON 2019 fulfilled the adopted comparison criteria. 263, 440 and 37 ozone profiles were compared over the periods DJFM 2013–2020, SON 2012–2018 and SON 2019, respectively. Plots were generated by using Python’s Matplotlib Library^63^.

Figure 2a,b show the significant seasonal changes that the vertical distribution of ozone exhibits over Antarctica. Within the layer from 12.5 to 22 km the concentration of ozone is on average about 30–50% lower during SON than during DJFM (except in the case of the Dumont d’Urville station that is often outside the polar vortex). Differences between balloon-borne and OMPS-LP-derived data change with the season, especially at altitudes lower than 22 km.

Most of the profiles exhibit a good agreement at altitudes higher than 22 km (see Fig. 2d–e,g–h). The *relative* mean bias errors (*rMBE*) are generally lower than ± 10% within the layer from 22 to 27.5 km except in the case of the Amundsen-Scott Station, which can be attributed to the long distance between the station and the orbit track. Within the layer from 12.5 to 22 km, the biases of the OMPS-LP-derived estimates are season-dependent.

As shown in Fig. 2g, OMPS-LP-derived estimates generally exhibit a negative mean bias error (up to about -20%) during DJFM (i.e. when the ozone concentration reach higher values). However, as shown in Fig. 2h, mean bias errors of OMPS-LP-derived estimates within the same layer (12.5–22 km) are not predominately negative during SON (i.e. when the Antarctic ozone depletion peaks); negative biases (up to about -10%) are apparent especially in the case of the Dumont d’Urville station (which is often outside the polar vortex) while positive biases (up to about + 15%) were found in the case of Marambio, and Davis stations (see Fig. 2h).

*RMSE* values tend to be larger (about 20%) in the lower stratosphere (12.5–17.5 km) and smaller (about 10%) within higher layers during DJFM (see Fig. 2g). *RMSE* values are significantly larger (even greater than 40%) during SON, especially within the layer from 12.5 to 22 km (see Fig. 2h). These relatively high *RMSE* values were expected for stations within the polar vortex (such as Amundsen-Scott) since during the ozone hole season (SON), the ozone concentration within this layer (12.5–22 km) can even fall below the detection limit of the ozonesondes^9^, which is about 10 ppbv^57^.

As shown in Figs. 2j,k, the correlation between the balloon-borne and OMPS-LP-derived ozone concentrations is generally high, especially during the SON period and at altitudes higher than 17.5 km, for which *R* values greater than 0.9 are generally found. An exception is again observed in the case of the Amundsen-Scott Station, which can be attributed to the long distance between the station and the orbit track. At lower altitudes (within the layer from 12.5 to 17.5 km), *R* values are somehow lower (but still higher than 0.55), regardless of the season. Lower correlations are nevertheless expected at lower altitudes, as satellite products tend to be more reliable at higher altitudes.

The differences between balloon-borne and OMPS-LP-derived profiles during the SSW event during SON 2019 are similar to those observed during other SON periods. As shown in Fig. 2 (third row), the profiles of *MBE, RMSE* and *R* computed during SON 2019 tend to follow those computed over the period SON 2012–2018. However, there are interesting differences. When comparing Fig. 2h,i, it can be observed that the *RMSE* values are, within the layer from 12.5 to 22 km (i.e. the most depleted during the ozone hole season), generally lower during SON 2019 than during SON 2012–2018. In fact, within this layer, *RMSE* values were closer to those observed over the period DJFM 2013–2020 (Fig. 2g) than to those observed over the period SON 2012–2018 (Fig. 2h). This is likely due to the fact that the SSW event during SON 2019 led to the smallest ozone hole observed in decades (i.e. ozone concentrations were closer to those registered during DJFM). Moreover, when comparing Figs. 2k,l, it can be observed that the *R* values were found to be lower during SON 2019 than during SON 2012–2018 within the layer from 12.5 to 15.5 km, which can be attributed to the relatively low number of ozonesondes that fulfilled the comparison criteria.

**Figure 3:**
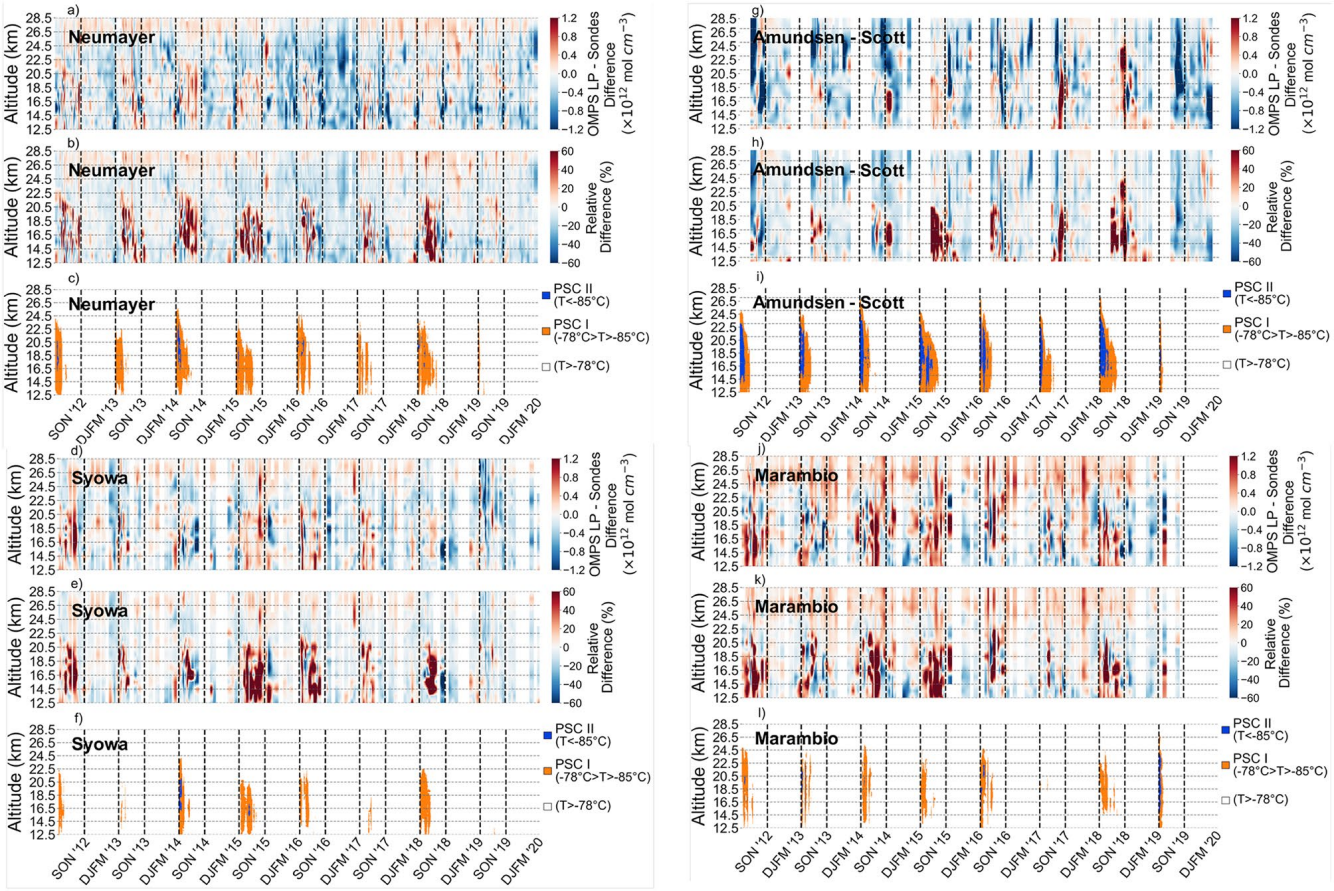
Heatmap of the differences (absolute and relative) between OMPS-LP-derived estimates and balloon-borne measurements; blank spaces indicates periods within which no sondes fulfilling the comparison criteria were available. Periods and altitudes at which the temperature favors the formation of polar stratospheric clouds (PSC) are shown below the heatmaps; temperature profiles from sondes were used. Dashed vertical lines separate different periods per year (DJFM and SON). (**a**–**c**) Neumayer; (**d**–**f**) Syowa; (**g**–**i**) Amundsen-Scott; and (**j**–**l**) Marambio. Plots were generated by using Python’s Matplotlib Library^63^.

A detailed representation of the relative difference between balloon-borne and OMPS-LP-derived data is shown in Fig. 3 for four stations, Neumayer (Fig. 3a–c), Syowa (Fig. 3d–f), Amundsen-Scott (South Pole, Fig. 3g–i) and Marambio (Fig. 3j–l). Periods and altitudes at which the temperature favors the formation of polar stratospheric clouds (PSC), type I and type II, are also shown. As expected, larger *relative* differences are observed during the ozone hole season (SON) when the lowest ozone concentrations occur. Once the ozone hole begins, the total ozone column drops rapidly at a rate of 3–5 DU per day, with nearly all of the ozone disappearing by late September within the layer from 14 to 22 km^58^, which generally led to large *relative* biases. Larger relative differences are also observed after the disappearance of the PSCs. This is attributable to the polar vortex that keeps the ozone-depleted air isolated from the surrounding ozone-rich air. The sharp meridional ozone gradient (from outside to inside of the polar vortex) may also negatively affect the agreement between balloon-borne and OMPS-LP-derived data, especially in the case of observations conducted close to the edge of the polar vortex. As expected, relative differences within the layer from 12.5 to 22 km peaked at 55% during the ozone hole season in 2015 (the greatest ozone hole of the last decade^59^). The prevalence of the red color in the heatmap within the layer from 12.5 to 22 km during the ozone hole season suggests that OMPS-LP-derived estimates tend to overestimate the ozone concentration in case of extremely low ozone abundances.

**Figure 4:**
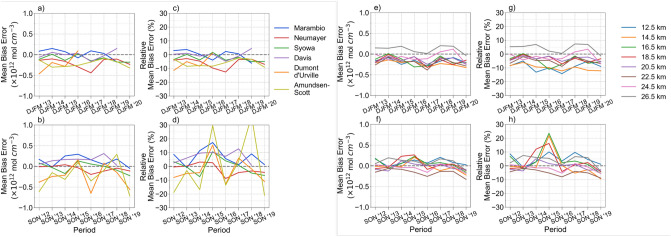
Time series of the absolute and relative mean bias errors (*MBE*) of OMPS-LP-derived estimates of the ozone (relative to balloon-borne data) computed over the DJFM period (first row) and over the SON period (second row). (**a**–**d**) per station; (**e**–**h**) per altitude. Plots were generated by using Python’s Matplotlib Library^63^.

Figure 4 presents the time series of the mean bias errors of OMPS-LP-derived estimates of ozone relative to balloon-borne data. OMPS-LP-derived estimates generally exhibit a negative mean bias error within the layer 12.5–22 km during DJFM (see Fig. 2g). The same negative mean bias error is apparent in Fig. 4g, which also suggests a small negative drift at lower altitudes (see for example the 14.5 km level). Prior efforts^32^ have tested OMPS-LP data (over 5.5 years) with profiles retrieved from the Odin Optical Spectrograph and InfraRed Imaging System (OSIRIS)^60^ and from the Aura Microwave Limb Sounder (MLS)^61^. Although Kramarova et al.^32^ found no significant drift when comparing OMPS-LP data with profiles retrieved from MLS, they did find a negative drift below 20 km when comparing OMPS-LP data with profiles retrieved from OSIRIS over Antarctic latitudes (90°–60° S). These results underline the challenges for the detection of drifts, especially over Antarctic latitudes (90°–60° S), where ozone is subjected to a significant year-to-year variability. In our case, the evaluation period (2012–2020) is likely too short for confirming/discarding a drift using balloon-borne measurements as reference.

During SON (when the ozone hole occurs), the relative mean bias errors were occasionally large (up to + 30%) within the layer from 12.5 to 22 km (see Fig. 4). This is likely related to the relatively low concentration of ozone within this layer over stations inside the polar vortex. Nevertheless, Fig. 4b shows that the absolute mean bias errors of OMPS-LP-derived estimates of the ozone (relative to balloon-borne data) are generally lower than 0.2 × 10^12^ molecules/cm^3^ (about 0.3 ppmv). Although according to Fig. 4b, the mean bias errors of OMPS-LP-derived estimates of ozone (relative to balloon-borne data) show some stability at the 0.3 ppmv level, it is worth highlighting that such a level may only allow for the detection of changes at time scales longer than decades.

In the Supplementary Material we present, for each launch site, the profiles of *MBE*, *RMSE* and *R* computed by comparing balloon-borne and OMPS-LP-derived data. In Table 2 we show the maximum and minimum values of the *MBE* and *RMSE* obtained from the corresponding profiles for each launch site. Also, for each altitude, we present in the Supplementary Material the correlations between the relative differences (between balloon-borne and OMPS-LP-derived data of ozone) and the following parameters: time difference between satellite readings and ozonesonde launch, distance to the station, SZA, SSA, tropopause altitude, surface reflectance, and the cloud height. These correlations may suggest potential issues that affect the retrieval algorithm. The following correlations were identified:

- significant correlations between the relative ozone differences and the surface reflectance were found during DJFM. The correlation is often positive for Amundsen-Scott (where *R* peaked at 0.62 at 27.5 km) but constantly negative for Syowa (where *R* peaked at − 0.55 at 20.5 km) and for Marambio (especially at altitudes higher than 17 km; in the case of Marambio, *R* peaked at − 0.40 at 24.5 km). Although correlations were found to be generally smaller during SON. Our results suggest that further efforts should be undertaken in future algorithm updates in order to minimize the sensitivity of the retrieved ozone profile to the underlying scene reflectance^32^;
- positive correlations between the relative ozone differences and the cloud height exists for Syowa during both SON and DJFM (in the case of Syowa, *R* peaked at 0.38 at 23.5 km during SON and at 0.34 at 21.5 km during DJFM); positive correlation were also found for Belgrano (where *R* peaked at 0.56 at 18.5 km during SON). During DJFM, negative correlations were found for Dumont d’Urville (where *R* peaked at − 0.53 at 23.5 km) as well as for Davis (where *R* peaked at − 0.34 at 22.5 km). Significant correlations are limited to only specific altitudes at other stations. The OMPS-LP algorithm retrieves ozone profiles from cloud top to 37.5 km; if no cloud is identified, the retrieval lower limit is set to 12.5 km. However, cloud detection over the bright Antarctic surfaces is particularly challenging in the ultraviolet and visible spectral range used by the OMPS-LP algorithm. The identified correlations between the relative ozone differences and the cloud height suggest that, in the case of Antarctica, there is still room for improvements in the cloud detection algorithm^62^;
- slightly positive correlations between the relative ozone differences and the *SZA* were found during SON for Dumont d’Urville, Amundsen-Scott and Belgrano. In contrast, correlations are consistently negative during DJFM for the majority of the stations e.g., for Neumayer and Syowa (especially at altitudes higher than 15.5 km), as well as for Marambio (where *R* peaked at − 0.53 at 23.5 km) and Davis (where *R* peaked at − 0.56 at 24.5 km). The correlation was also negative during SON for Syowa (where *R* peaked at − 0.44 at 25.5 km). The dependence on the SZA is typical for satellite products of the total ozone column and often emerges from validation efforts based on ground-based observations (as ground observations are more uncertain for low solar elevations)^25^. However, the influence of the SZA on ozone differences was unexpected in our case because ozonesonde data do not depend on the SZA. Therefore, straightforward conclusions cannot be drawn on this issue;
- positive correlations between the relative ozone differences and the tropopause altitude were found, mainly at altitudes lower than 20 km. This correlation was particularly clear during SON in the case of Amundsen-Scott and Belgrano II where *R* peaked for both stations at 15.5 km at 0.61 and 0.78, respectively. Nevertheless, conclusions on this issue remain challenging since results for Belgrano II are based on few ozone data pairs (15 over the period 2012–2018) while for Amundsen-Scott the distance between satellite observations and ozonesondes is on average about two times larger than for other stations;
- negative correlations between relative ozone differences and the SSA were detected especially during DJFM at the upper altitudes; e.g., during DJFM for Neumayer (where *R* peaked at − 0.34 at 23.5 km), Marambio (where *R* peaked at − 0.50 at 23.5 km), Syowa (where *R* peaked at − 0.30 at 23.5 km), and Davis (where *R* peaked at − 0.55 at 24.5 km).

**Table 2:** Mean Bias Error (*MBE*) and Root Mean Square Error (*RMSE*) over the periods: DJFM 2013–2020, SON 2012–2018 and SON 2019.

Station	MBE (%)	MBE (× 10^12^ mol cm^-3^)	max MBE (× 10^12^ mol cm^-3^)	max MBE—Altitude (km)	min MBE (× 10^12^ mol cm^-3^)	min MBE—Altitude (km)	RMSE (%)	RMSE (× 10^12^ mol cm^-3^)	max RMSE (× 10^12^ mol cm^-3^)	max RMSE—Altitude (km)	min RMSE (× 10^12^ mol cm^-3^)	min RMSE—Altitude (km)	*R*
**DJFM**													
Marambio	1.1	0.04	0.39	25.5	− 0.22	12.5	14	0.44	0.58	17.5	0.25	27.5	0.92
Belgrano II	–	–	–	–	–	–	–	–	–	–	–	–	–
Neumayer	− 5.8	− 0.20	0.06	27.5	− 0.35	15.5	13	0.42	0.56	15.5	0.22	27.5	0.93
Syowa	− 3.0	− 0.10	0.14	26.5	− 0.23	22.5	11	0.37	0.51	19.5	0.25	27.5	0.94
Davis	− 1.1	− 0.04	0.26	26.5	− 0.28	14.5	13	0.42	0.61	14.5	0.29	27.5	0.92
Dumont d’Urville	− 6.2	− 0.22	0.10	25.5	− 0.45	16.5	15	0.53	0.77	17.5	0.27	27.5	0.91
Amundsen-Scott	− 6.7	− 0.22	0.03	12.5	− 0.38	21.5	16	0.54	0.73	17.5	0.28	27.5	0.90
Escudero	–	–	–	–	–	–	–	–	–	–	–	–	–
**SON**													
Marambio	5.7	0.15	0.30	25.5	− 0.01	21.5	27	0.70	1.05	18.5	0.31	27.5	0.86
Belgrano II	0.3	0.01	0.19	20.5	− 0.43	14.5	24	0.47	0.64	14.5	0.29	16.5	0.94
Neumayer	− 2.7	− 0.06	0.11	27.5	− 0.22	23.5	21	0.44	0.65	15.5	0.23	26.5	0.93
Syowa	0.4	0.01	0.14	20.5	− 0.19	22.5	21	0.47	0.72	16.5	0.18	27.5	0.92
Davis	5.6	0.14	0.40	26.5	− 0.02	17.5	26	0.68	1.12	18.5	0.37	14.5	0.88
Dumont d’Urville	− 6.1	− 0.24	0.06	12.5	− 0.66	20.5	23	0.91	1.29	16.5	0.47	27.5	0.86
Amundsen-Scott	− 7.5	− 0.17	0.15	12.5	− 0.43	24.5	34	0.79	1.01	23.5	0.36	12.5	0.85
Escudero	–	–	–	–	–	–	–	–	–	–	–	–	–
**SON 2019**													
Marambio	–	–	–	–	–	–	–	–	–	–	–	–	–
Belgrano II	− 6.6	− 0.16	0.06	20.5	− 0.42	15.5	11	0.28	0.45	15.5	0.09	27.5	0.97
Neumayer	− 4.5	− 0.11	0.06	21.5	− 0.48	15.5	14	0.35	0.65	15.5	0.25	25.5	0.94
Syowa	− 7.0	− 0.22	0.01	20.5	− 0.46	22.5	18	0.58	1.01	19.5	0.28	12.5	0.88
Davis	–	–	–	–	–	–	–	–	–	–	–	–	–
Dumont d’Urville	–	–	–	–	–	–	–	–	–	–	–	–	–
Amundsen-Scott	− 22	− 0.72	0.21	12.5	− 1.30	18.5	34	1.10	1.99	19.5	0.25	13.5	0.78
Escudero	1.6	0.04	0.54	27.5	− 0.42	18.5	20	0.51	0.86	15.5	0.29	24.5	0.89

Maximum and minimum values of the *MBE* and *RMSE* profiles are also shown. Since few profiles were available, data are not shown for some stations.

Distance and time differences were found to marginally affect the relative ozone differences (except for Dumont d’Urville and Belgrano for specific altitude ranges and seasons).

## Summary and conclusions

Ozone plays an important role in the radiative budget, affecting the atmospheric circulation and climate. Even small variations in the distributions of trace gases, like ozone, can significantly impact the radiative forcing of Earth’s climate and are of key importance for understanding climate change. Therefore, a better understanding of the transport related processes that control the concentrations of radiatively and chemically active species like ozone is of great interest.

Predicting the radiative forcing due to stratospheric ozone recovery and related processes during this century requires detecting changes in the vertical distribution of ozone. However, trend detection in the case of ozone is complicated by the climate variability (in the season 2019–2020 one of the smallest ozone holes occurred over Antarctica and one of the largest ozone loss events occurred over the Arctic). Overcoming these challenges requires improving the accuracy of satellite-derived estimates. In this endeavor, the validation of satellite products plays a key role.

Here, we have carried out a systematic comparison between “last state” OMPS-LP ozone profiles (version 2.5 algorithm) and balloon-borne measurements of the ozone abundance gathered by ECC-type ozonesondes. The OMPS-LP instrument, aboard the Suomi NPP satellite, provides estimates of both the total ozone column and ozone profiles since October 2011. The balloons were launched from 8 Antarctic stations over the period 2012–2020. Comparisons focused on the layer from 12.5 to 27.5 km and were conducted over two periods: SON and DJFM.

We found that most of the profiles exhibit a good agreement within the layer from 22 to 27.5 km, within which *MBE* values are generally lower than ± 10% (except in the case of the Amundsen-Scott Station). Within the layer from 12.5 to 22 km, the biases of the OMPS-LP-derived estimates are season-dependent; *MBE* values remain in the same range (± 10%) as at higher altitudes during the ozone hole season (SON), but *MBE* values become predominantly negative (up to about − 20%) during DJFM (i.e. when the ozone concentration reaches higher values). Nevertheless, we found that relative to balloon-borne data, *MBE* values of OMPS-derived Antarctic ozone profiles are generally less than 0.3 ppmv.

We also found that, during DJFM, *RMSE* values tend to be larger (about 20%) in the lower stratosphere (12.5–17.5 km) and smaller (about 10%) within higher layers (17.5–27.5 km). However, during the ozone hole season (SON), *RMSE* values exhibit larger figures (even greater than 40%) within the layer from 12.5 to 22 km, especially in the case of stations within the polar vortex (such as Amundsen-Scott).

Our results suggest that the differences between balloon-borne and OMPS-derived Antarctic ozone profiles are generally within the bounds defined by the uncertainties of both satellite-derived and balloon-borne data. The associated uncertainty of ECC ozonesonde measurements is about 10% in the troposphere and 5% in the stratosphere up to 10 hPa, and 5–25% between 10 and 3 hPa^49–55^, while the expected precision for ozone retrievals is better than 20% for elevations lower than 25 km and 5–10% for elevations from 25–50 km^38^.

The total ozone over Antarctic latitudes (90°-60°S) in the months of September and October is increasing at a rate of about 6–8% per decade (see Fig. 4.15 in Scientific Assessment of Ozone Depletion^10^). If the Antarctic stratospheric ozone returns to 1980 values in the 2060s as projected by WMO^10^, ozone concentrations at 20 km should increase in the next decades at a rate in the range from 0.2 to 0.3 ppmv/decade. This means that, as the ozone hole closes, the inter-decadal increases in the ozone concentration expected in the upcoming decades at altitudes of about 20 km over Antarctica are similar to the mean bias error of OMPS-derived ozone concentrations (generally less than 0.3 ppmv relative to balloon-borne measurements).

Relative to balloon-borne measurements, satellite-derived data exhibit no major drift, but the evaluation period (2012–2020) is likely too short for confirming/discarding a drift. Significant correlations were found between the satellite estimates biases and the tropopause altitude, the cloud height, and the surface reflectance. These correlations suggest that further efforts to minimize the retrieval errors should focus on improving the sensitivity of the algorithm to the underlying Antarctic conditions.

## Supplementary Information

Supplementary Information.

## Data Availability

Satellite-derived ozone profiles were obtained from the NASA Goddard Space Flight Center web site (Aura Validation data center): https://gs614-avdc1-pz.gsfc.nasa.gov/pub/data/satellite/Suomi_NPP/L2OVP/LP-L2-O3-DAILY/. Balloon-borne ozonesonde data were obtained from the World Ozone and UV Data Center (WOUDC: https://woudc.org/data/explore.php?lang=en) and the Network for the Detection of Atmospheric Composition Change (NDACC: https://www.ndaccdemo.org). Additional datasets and codes are available from the corresponding author on reasonable request.
