# Supplementary material for: Evaluation of Antarctic Ozone Profiles derived from OMPS-LP by using Balloon-borne Ozonesondes
Source: Sci Rep. 2021 Feb 22;11:4288. doi: 10.1038/s41598-021-81954-6 (PMC7900121; doi:10.1038/s41598-021-81954-6)
Supplement: Supplementary file 1 — Supplementary Information. [file 41598_2021_81954_MOESM1_ESM.pdf]

Supplementary Material

Evaluation of Antarctic Ozone Profiles derived from OMPS-LP by using Balloon-borne Ozonesondes

Edgardo Sepúlveda[1], Raul R. Cordero[1,*], Alessandro Damiani[2], Sarah Feron[1,3,§], Jaime Pizarro[1], Felix Zamorano[4], Rigel Kivi[5], Ricardo Sánchez[6], Margarita Yela[7], Julien Jumelet[8], Alejandro Godoy[6], Jorge Carrasco[4], Juan S. Crespo[9], Gunther Seckmeyer[10], Jose A. Jorquera[1], Juan M. Carrera[1], Braulio Valdevenito[1], Sergio Cabrera[11], Alberto Redondas[12], Penny M. Rowe[1,13]

1 Universidad de Santiago de Chile. Av. B. O'Higgins 3363, Santiago, Chile.
2 Center of Environmental Remote Sensing, Chiba University, Chiba, Japan.
3 Department of Earth System Science, Stanford University, Stanford, CA, 94305-2210, USA.
4 University of Magallanes, Punta Arenas, Chile.
5 Finnish Meteorological Institute (FMI), Space and Earth Observation Centre, Sodankylä, Finland.
6 Servicio Meteorológico Nacional, Buenos Aires, Argentina.
7 Instituto Nacional de Técnica Aeroespacial (INTA), Madrid, Spain.
8 LATMOS/IPSL, Sorbonne Université, UVSQ, CNRS, Paris, France.
9 Dirección Meteorológica de Chile, Santiago, Chile.
10 Leibniz Universität Hannover, Herrenhauser Strasse 2, Hannover, Germany.
11 Instituto de Ciencias Biomédicas, Universidad de Chile, Santiago, Chile.
12 Izaña Atmospheric Research Center (IARC), State Meteorological Agency (AEMET), Santa Cruz de Tenerife, Spain.
13 NorthWest Research Associates, Redmond, WA, USA.

Corresponding authors:
* Email: raul.cordero@usach.cl
Tel: +56(9)97352030
§ Email: sferon@stanford.edu
Tel: +1 (650) 422-9098

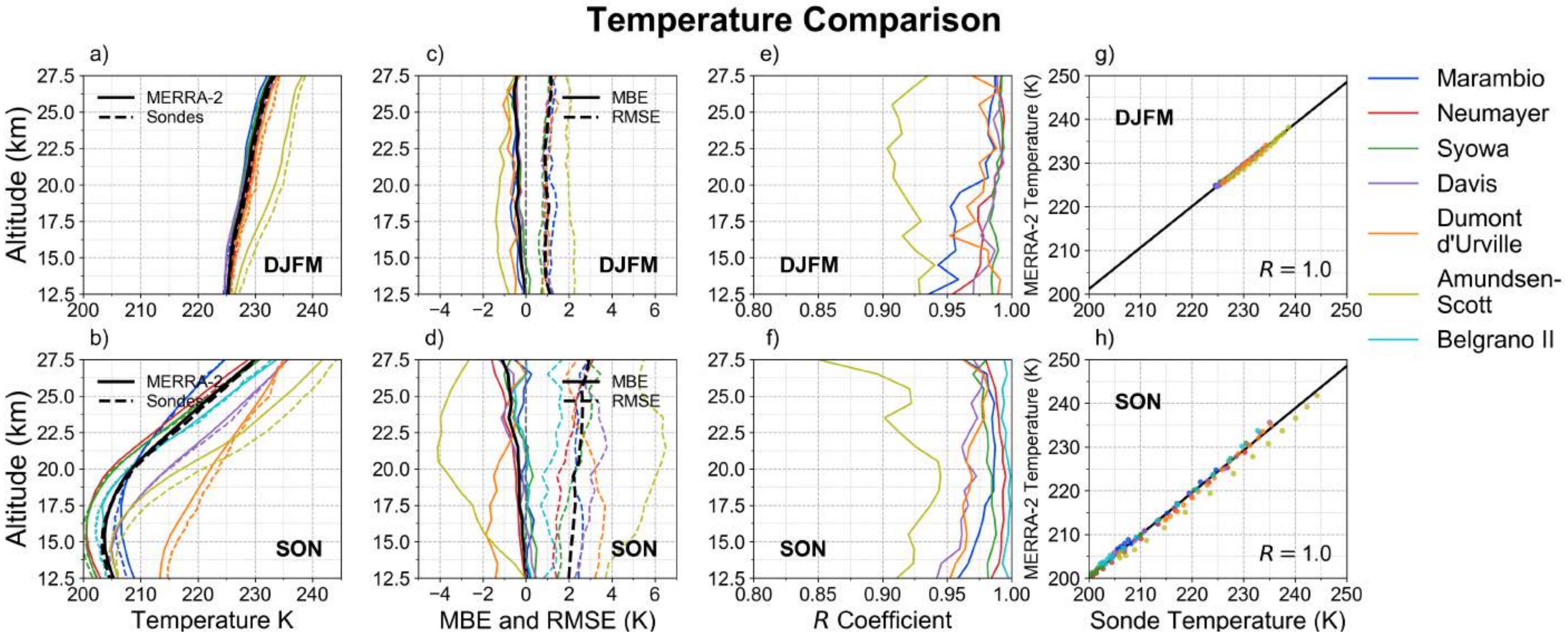

Fig. S1. Comparison between MERRA-2 reanalysis data of the temperature (embedded in OMPS-LP dataset) and balloon-borne measurements of the temperature over the period DJFM 2013-2020 (first row), and over the period SON 2012-2018 (second row).

a-b) Mean profiles measured at each launch site. The solid line stands for the MEAN computed from MERRA-2 temperatures and the dashed line stands for the MEAN computed from balloon-borne measurements of the temperature. 263 and 440 temperature profiles were compared over the periods the DJFM 2013-2020 and SON 2012-2018, respectively;

c-d) Mean Bias Error (*MBE*, solid line) and Root Mean Square Error (*RMSE*, dashed line) relative to balloon-borne temperature data;

e-f) *R* profile;

g-h) Scatter plot (the correlation coefficient is shown in the plot).

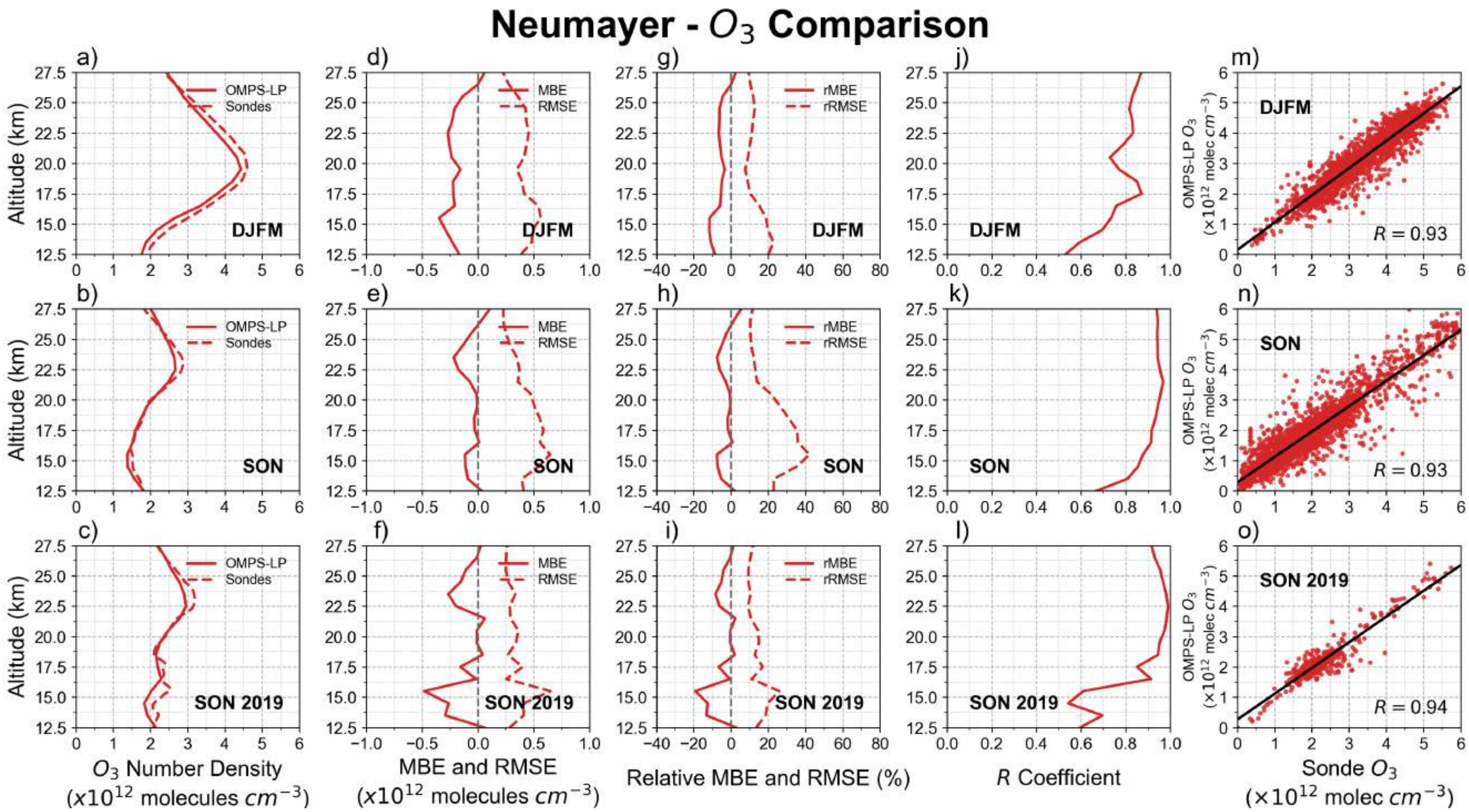

Fig. S2. Comparison between OMPS-LP-derived and balloon-borne data of ozone over the period DJFM 2013-2020 (first row), over the period SON 2012-2018 (second row) and over the period SON 2019 (third row) for Neumayer Station.

a-c) Mean profiles. The solid line stands for the MEAN computed from OMPS-LP-derived data of ozone and the dashed line stands for the MEAN computed from balloon-borne measurements of ozone. 114, 179 and 19 ozone profiles were compared over the periods DJFM 2013-2020, SON 2012-2018 and SON 2019, respectively;
d-f) Mean Bias Error (*MBE*, solid line) and Root Mean Square Error (*RMSE*, dashed line) relative to balloon-borne ozone data;
g-i) Relative Mean Bias Error (*MBE*, solid line) and Relative Root Mean Square Error (*RMSE*, dashed line);
j-l) *R* profile;
m-o) Scatter plot (the correlation coefficient *R* is shown in the plot).

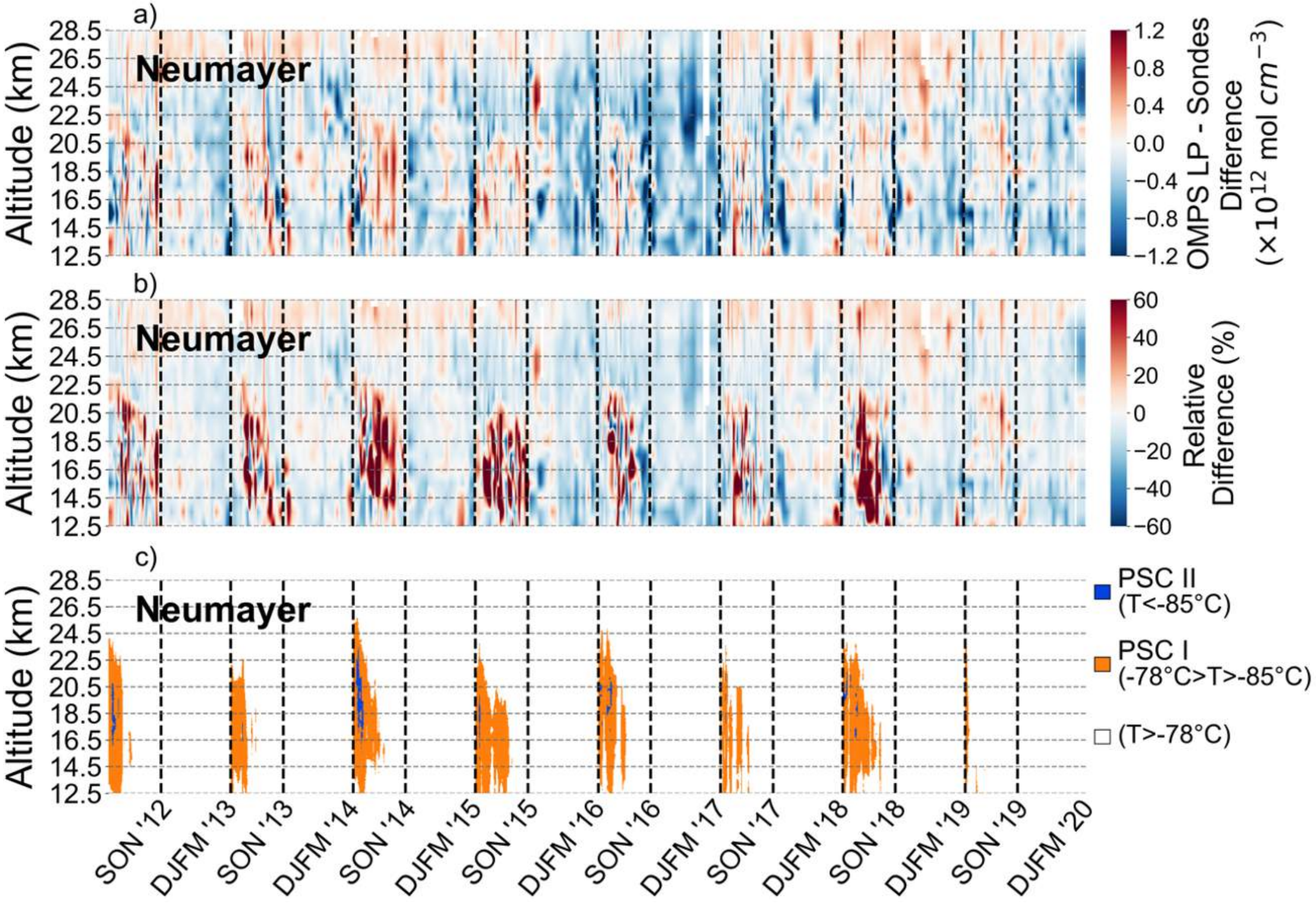

Fig. S3. Heatmap of the differences (absolute and relative) between OMPS-LP-derived estimates and balloon-borne measurements; blank spaces indicates periods within which no sondes fulfilling the comparison criteria were available. Periods and altitudes at which the temperature favors the formation of polar stratospheric clouds (PSC) are shown below the heatmaps; temperature profiles from sondes were used. Dashed lines separate different periods (DJFM and SON).

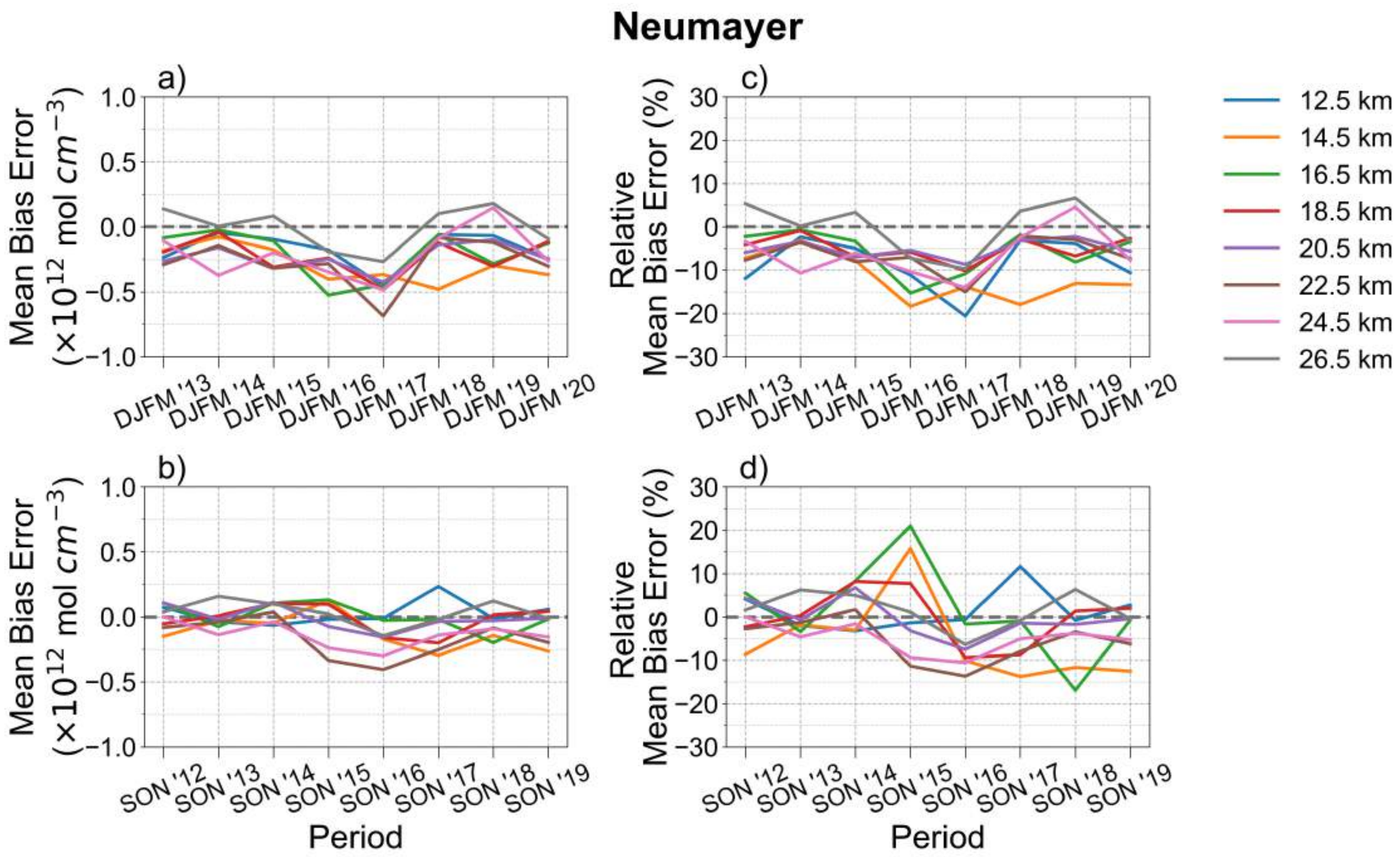

Fig. S4. Time series of the absolute and relative mean bias errors (*MBE*) of OMPS-LP-derived estimates of the ozone (relative to balloon-borne data) computed for Neumayer Station, over the DJFM period (first row) and over the SON period (second row).

Table S1. *R* coefficients between several OMPS-LP parameters (SZA, SSA, Reflectance, etc.) and the relative differences (between the OMPS-LP-derived estimates and the balloon-borne measurements of ozone) over the period DJFM 2013-2020. *R* coefficients higher than 0.2 and lower than -0.2 are in bold.

NEUMAYER - DJFM							
Altitude (km)	Time Difference	Distance	SZA	SSA	Tropopause	Surface Reflectance	Cloud Height
12.5	0.12	0.07	0.11	0.07	0.14	**0.28**	0.12
13.5	-0.04	0.06	0.12	0.09	0.08	**0.29**	0.14
14.5	0.04	0.00	0.00	-0.04	-0.05	0.18	0.09
15.5	-0.10	0.00	-0.15	-0.14	0.09	0.13	0.00
16.5	-0.08	-0.02	-0.09	-0.07	-0.16	0.04	0.17
17.5	0.04	0.14	-0.05	-0.03	-0.01	0.11	0.01
18.5	0.02	-0.14	**-0.23**	**-0.21**	**0.21**	-0.04	0.04
19.5	-0.08	-0.01	**-0.21**	-0.19	0.06	-0.11	0.06
20.5	0.01	0.01	-0.13	-0.12	0.02	-0.05	0.04
21.5	**-0.21**	0.09	-0.13	-0.15	-0.01	-0.11	0.04
22.5	-0.07	0.14	-0.20	**-0.22**	0.09	-0.02	0.11
23.5	-0.12	0.11	**-0.31**	**-0.34**	0.11	-0.11	0.13
24.5	**-0.24**	-0.02	**-0.30**	**-0.31**	0.09	-0.06	0.11
25.5	**-0.27**	0.07	**-0.29**	**-0.30**	0.02	-0.14	0.20
26.5	**-0.27**	0.03	**-0.27**	**-0.28**	0.04	-0.14	0.14
27.5	-0.09	-0.01	-0.20	**-0.20**	0.03	-0.12	0.15
Full Altitude Range	-0.07	0.03	-0.10	-0.11	0.04	0.05	0.09

Table S2. *R* coefficients between several OMPS-LP parameters (SZA, SSA, Reflectance, etc.) and the relative differences (between the OMPS-LP-derived estimates and the balloon-borne measurements of ozone) over the period SON 2012-2018. *R* coefficients higher than 0.2 and lower than -0.2 are in bold.

NEUMAYER - SON							
Altitude (km)	Time Difference	Distance	SZA	SSA	Tropopause	Surface Reflectance	Cloud Height
12.5	0.10	-0.01	-0.12	-0.11	-0.04	-0.09	0.14
13.5	0.04	0.01	-0.04	-0.04	0.12	0.06	-0.02
14.5	-0.10	0.19	-0.11	-0.13	0.07	0.20	-0.01
15.5	-0.04	0.07	0.00	-0.03	0.03	0.17	-0.01
16.5	0.04	-0.03	0.09	0.06	0.03	0.12	0.05
17.5	0.12	0.00	0.02	0.01	-0.01	-0.06	-0.01
18.5	0.04	-0.01	-0.03	-0.05	-0.03	0.02	0.04
19.5	0.03	0.01	-0.04	-0.06	-0.13	0.07	-0.08
20.5	0.01	-0.04	0.06	0.06	0.07	**0.24**	0.02
21.5	-0.02	0.00	**0.25**	**0.24**	0.07	**0.24**	-0.02
22.5	0.00	-0.02	**0.23**	**0.22**	0.00	-0.02	0.03
23.5	-0.04	-0.08	-0.18	-0.17	-0.19	-0.14	-0.17
24.5	-0.03	-0.13	-0.19	-0.19	-0.09	0.10	-0.05
25.5	-0.05	-0.04	**-0.22**	**-0.21**	-0.17	0.06	-0.01
26.5	-0.12	0.02	-0.16	-0.16	-0.16	0.07	-0.06
27.5	-0.16	0.04	-0.03	-0.03	0.02	0.18	-0.01
Full Altitude Range	0.01	0.01	-0.01	-0.02	-0.01	0.06	-0.01

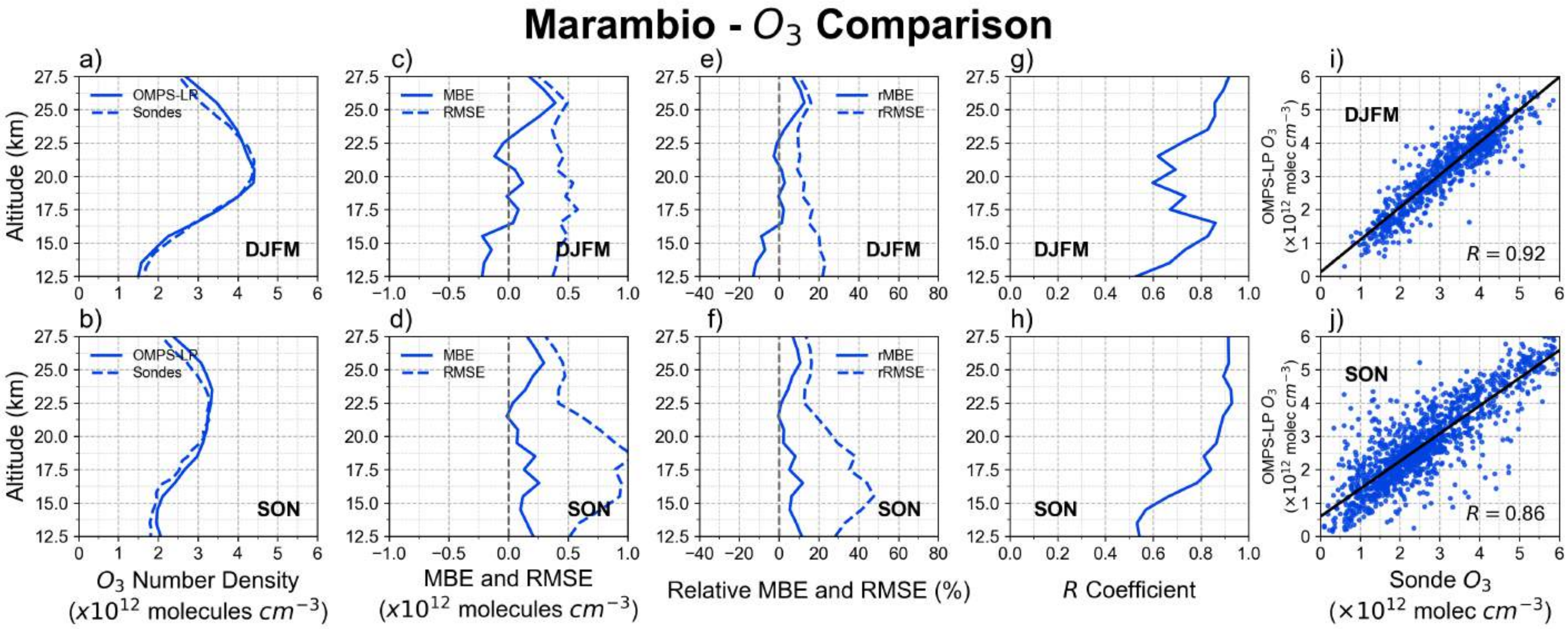

Fig. S5. Comparison between OMPS-LP-derived and balloon-borne data of ozone over the period DJFM 2013-2019 (first row), and over the period SON 2012-2018 (second row) for Marambio Station.

a-b) Mean profiles. The solid line stands for the MEAN computed from OMPS-LP-derived estimates of ozone and the dashed line stands for the MEAN computed from balloon-borne measurements of ozone. 47 and 81 ozone profiles were compared over the periods DJFM 2013-2019 and SON 2012-2018, respectively;
c-d) Mean Bias Error (*MBE*, solid line) and Root Mean Square Error (*RMSE*, dashed line) relative to balloon-borne ozone data;
e-f) Relative Mean Bias Error (*MBE*, solid line) and Relative Root Mean Square Error (*RMSE*, dashed line);
g-h) *R* profile;
i-j) Scatter plot (the correlation coefficient is shown in the plot).

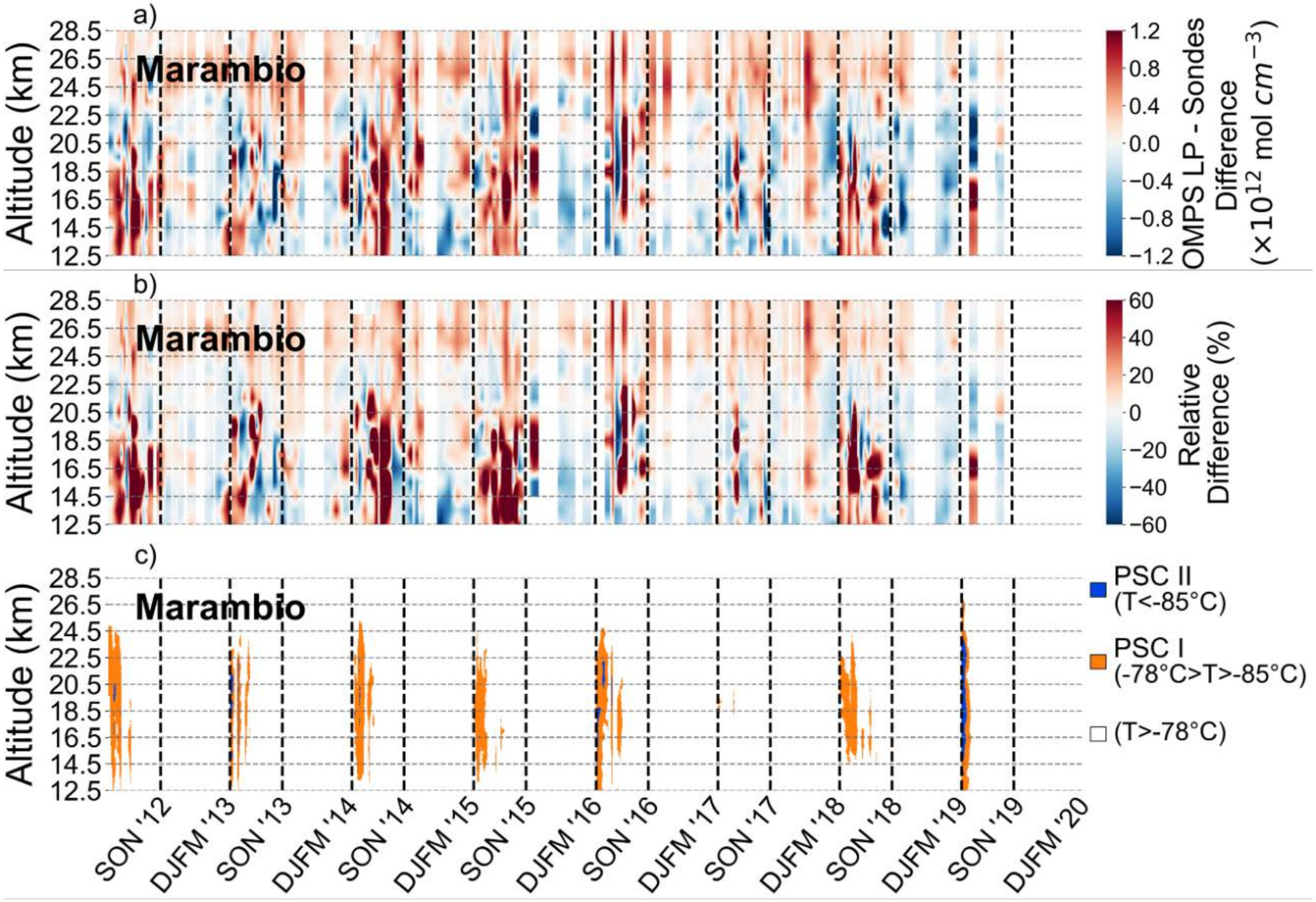

Fig. S6. Heatmap of the differences (absolute and relative) between OMPS-LP-derived estimates and balloon-borne measurements; blank spaces indicates periods within which no sondes fulfilling the comparison criteria were available. Periods and altitudes at which the temperature favors the formation of polar stratospheric clouds (PSC) are shown below the heatmaps; temperature profiles from sondes were used. Dashed lines separate different periods (DJFM and SON).

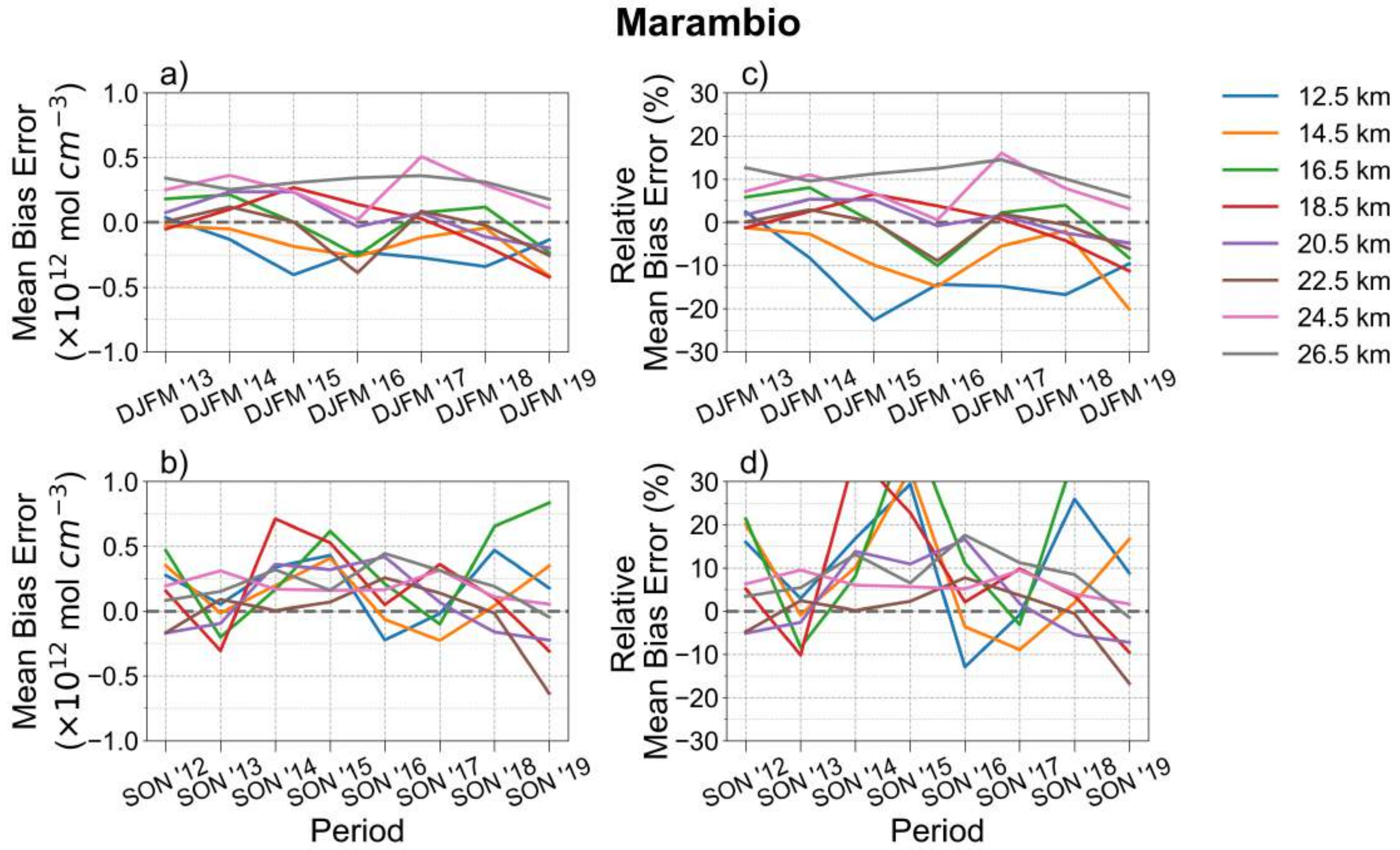

Fig. S7. Time series of the absolute and relative mean bias errors (*MBE*) of OMPS-LP-derived estimates of the ozone (relative to balloon-borne data) computed for Marambio Station, over the DJFM period (first row) and over the SON period (second row).

Table S3. *R* coefficients between several OMPS-LP parameters (SZA, SSA, Reflectance, etc.) and the relative differences (between the OMPS-LP-derived estimates and the balloon-borne measurements of ozone) over the period DJFM 2013-2020. *R* coefficients higher than 0.2 and lower than -0.2 are in bold.

MARAMBIO - DJFM							
Altitude (km)	Time Difference	Distance	SZA	SSA	Tropopause	Surface Reflectance	Cloud Height
12.5	0.16	0.18	**0.27**	**0.28**	-0.11	0.15	-0.11
13.5	**0.25**	-0.20	**0.23**	**0.23**	0.03	0.19	0.12
14.5	0.03	0.01	0.18	0.16	-0.19	**0.35**	**0.23**
15.5	0.00	0.12	0.02	0.04	**-0.23**	**0.40**	**0.37**
16.5	0.16	0.04	-0.06	-0.12	**-0.21**	0.04	0.07
17.5	0.17	-0.05	-0.18	**-0.23**	-0.14	-0.06	-0.02
18.5	0.12	-0.05	-0.14	-0.18	-0.09	**-0.21**	-0.08
19.5	0.07	-0.03	**-0.21**	**-0.25**	**-0.22**	**-0.29**	0.01
20.5	-0.04	-0.04	**-0.24**	**-0.24**	**-0.31**	**-0.24**	-0.11
21.5	0.03	0.12	-0.11	-0.08	-0.18	**-0.20**	-0.02
22.5	-0.08	-0.01	**-0.39**	**-0.35**	**-0.25**	**-0.30**	-0.17
23.5	**-0.21**	**-0.32**	**-0.53**	**-0.50**	**-0.39**	**-0.39**	-0.15
24.5	-0.01	**-0.25**	**-0.34**	**-0.33**	**-0.24**	**-0.40**	-0.14
25.5	0.09	-0.07	0.10	0.09	-0.15	**-0.28**	-0.02
26.5	0.19	-0.09	0.12	0.16	0.06	-0.17	0.19
27.5	0.01	-0.14	0.08	0.11	-0.08	-0.19	0.04
Full Altitude Range	0.07	-0.03	-0.04	-0.05	-0.13	-0.04	0.03

Table S4. *R* coefficients between several OMPS-LP parameters (SZA, SSA, Reflectance, etc.) and the relative differences (between the OMPS-LP-derived estimates and the balloon-borne measurements of ozone) over the period SON 2012-2018. *R* coefficients higher than 0.2 and lower than -0.2 are in bold.

MARAMBIO - SON							
Altitude (km)	Time Difference	Distance	SZA	SSA	Tropopause	Surface Reflectance	Cloud Height
12.5	0.13	0.04	0.05	0.04	0.02	0.10	-0.16
13.5	**0.23**	0.01	-0.05	-0.07	-0.02	0.06	-0.09
14.5	**0.29**	0.01	0.03	0.02	-0.04	0.11	-0.06
15.5	0.16	-0.05	0.08	0.06	0.11	0.14	-0.16
16.5	-0.02	0.10	0.04	0.04	**0.22**	0.15	-0.02
17.5	0.03	-0.13	-0.03	-0.04	0.09	0.03	-0.09
18.5	0.07	-0.12	0.08	0.06	0.12	0.04	-0.14
19.5	-0.01	0.01	-0.01	-0.01	0.08	0.01	-0.04
20.5	-0.10	0.07	-0.01	-0.02	0.01	0.09	0.08
21.5	-0.11	0.20	-0.12	-0.13	-0.04	-0.03	-0.01
22.5	-0.02	0.06	**-0.34**	**-0.33**	-0.15	**-0.26**	-0.04
23.5	-0.13	0.08	**-0.37**	**-0.35**	**-0.27**	**-0.27**	-0.01
24.5	-0.04	0.16	-0.16	-0.15	-0.15	**-0.21**	-0.11
25.5	-0.14	0.20	0.06	0.06	0.04	-0.08	0.07
26.5	-0.03	0.18	0.09	0.09	0.06	0.01	**0.21**
27.5	-0.04	0.08	-0.07	-0.07	0.07	-0.06	**0.23**
Full Altitude Range	0.04	0.00	0.00	-0.01	0.05	0.04	-0.05

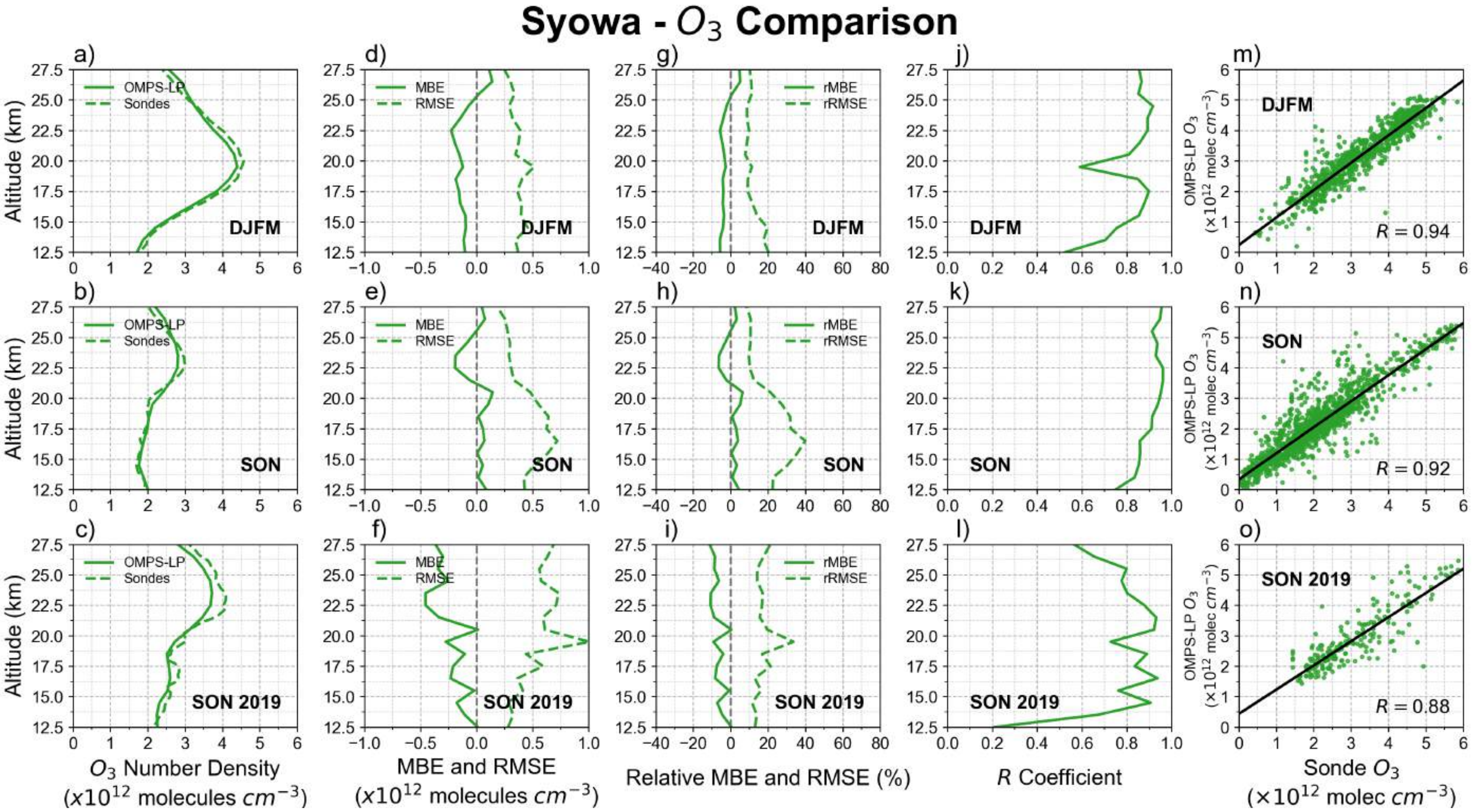

Fig. S8. Comparison between OMPS-LP-derived and balloon-borne data of ozone over the period DJFM 2013-2020 (first row), over the period SON 2012-2018 (second row) and over the period SON 2019 (third row) for Syowa Station.

a-c) Mean profiles. The solid line stands for the MEAN computed from OMPS-LP-derived estimates of ozone and the dashed line stands for the MEAN computed from balloon-borne measurements of ozone. 52, 83 and 13 ozone profiles were compared over the periods DJFM 2013-2020, SON 2012-2018 and SON 2019, respectively;

d-f) Mean Bias Error (*MBE*, solid line) and Root Mean Square Error (*RMSE*, dashed line) relative to balloon-borne ozone data;

g-i) Relative Mean Bias Error (*MBE*, solid line) and Relative Root Mean Square Error (*RMSE*, dashed line);

j-l) *R* profile;

m-o) Scatter plot (the correlation coefficient is shown in the plot).

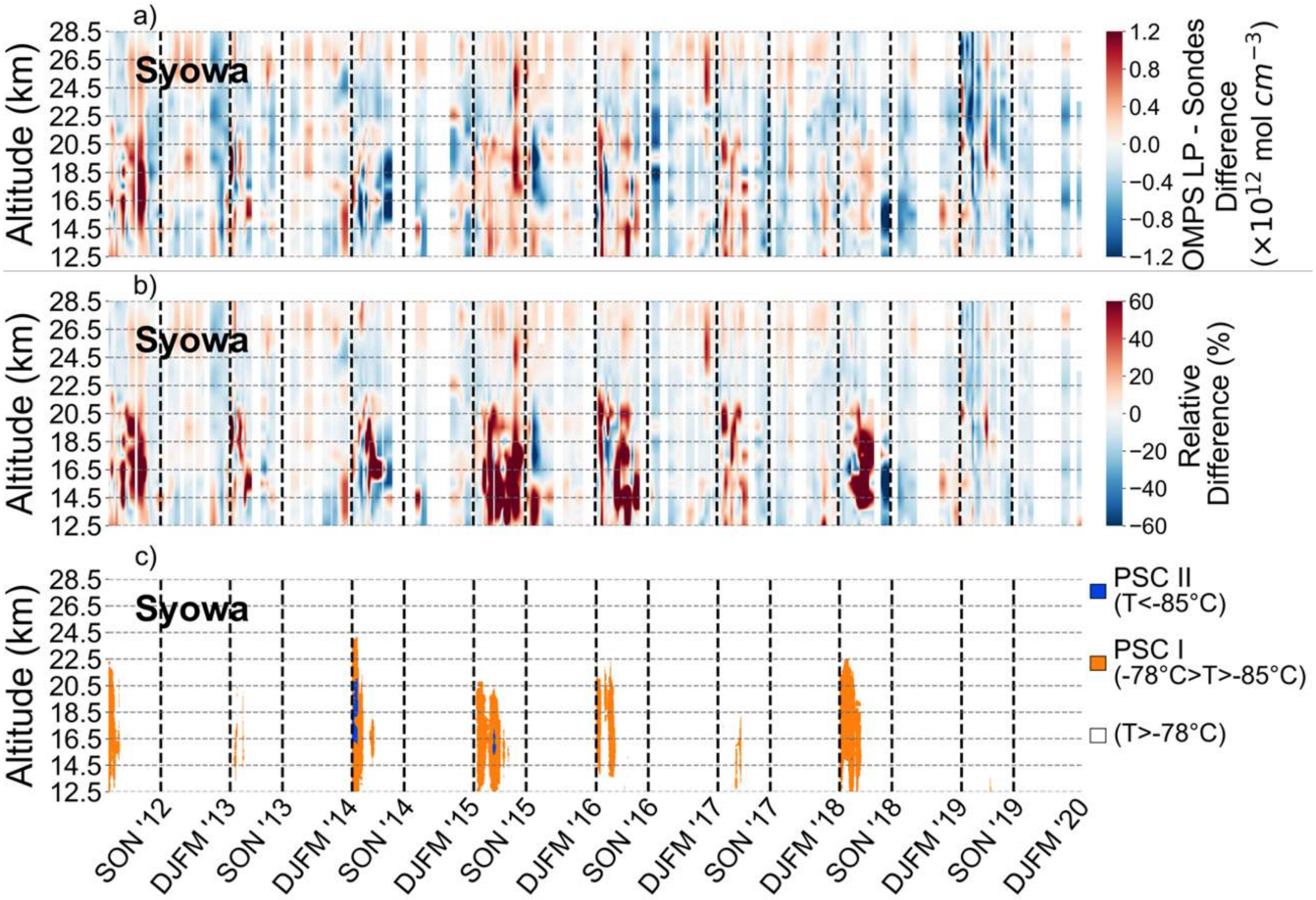

Fig. S9. Heatmap of the differences (absolute and relative) between OMPS-LP-derived estimates and balloon-borne measurements; blank spaces indicates periods within which no sondes fulfilling the comparison criteria were available. Periods and altitudes at which the temperature favors the formation of polar stratospheric clouds (PSC) are shown below the heatmaps; temperature profiles from sondes were used. Dashed lines separate different periods (DJFM and SON).

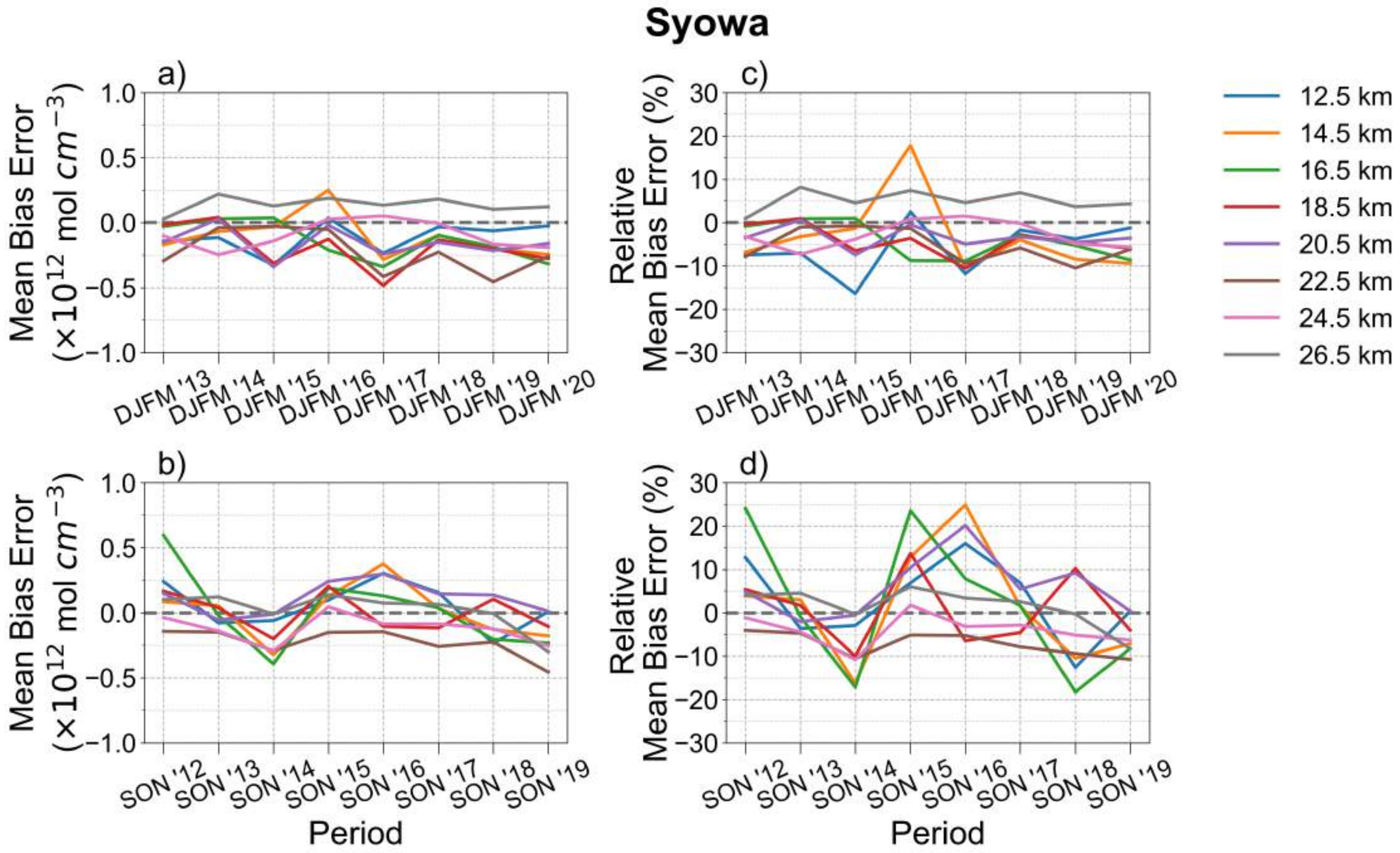

Fig. S10. Time series of the absolute and relative mean bias errors (*MBE*) of OMPS-LP-derived estimates of the ozone (relative to balloon-borne data) computed for Syowa Station, over the DJFM period (first row) and over the SON period (second row).

Table S5. *R* coefficients between several OMPS-LP parameters (SZA, SSA, Reflectance, etc.) and the relative differences (between the OMPS-LP-derived estimates and the balloon-borne measurements of ozone) over the period DJFM 2013-2020. *R* coefficients higher than 0.2 and lower than -0.2 are in bold.

SYOWA - DJFM							
Altitude (km)	Time Difference	Distance	SZA	SSA	Tropopause	Surface Reflectance	Cloud Height
12.5	**-0.20**	-0.04	**0.25**	**0.23**	-0.12	**0.32**	**-0.20**
13.5	-0.08	0.04	0.11	0.08	0.07	**0.35**	-0.04
14.5	0.03	0.08	-0.01	-0.04	0.10	0.18	-0.07
15.5	-0.05	**0.20**	0.18	0.18	0.07	0.06	0.12
16.5	-0.13	0.11	**0.28**	**0.27**	-0.18	**-0.21**	-0.01
17.5	0.01	0.03	0.15	0.17	-0.11	**-0.38**	0.09
18.5	0.02	-0.03	-0.01	0.00	-0.10	**-0.45**	0.14
19.5	0.08	-0.06	-0.11	-0.11	-0.03	**-0.50**	0.13
20.5	0.12	-0.06	-0.09	-0.05	0.15	**-0.55**	**0.24**
21.5	0.08	-0.12	-0.09	-0.06	0.11	**-0.51**	**0.34**
22.5	**0.41**	0.04	**-0.21**	**-0.23**	0.16	-0.12	0.12
23.5	**0.33**	0.10	**-0.31**	**-0.30**	**0.21**	-0.18	**0.23**
24.5	**0.28**	0.17	**-0.23**	**-0.21**	**0.23**	-0.17	**0.30**
25.5	**0.24**	**0.20**	**-0.25**	**-0.24**	**0.23**	-0.19	0.20
26.5	**0.21**	0.12	**-0.25**	**-0.23**	**0.28**	**-0.25**	**0.26**
27.5	0.12	-0.04	-0.07	-0.05	0.11	**-0.41**	0.10
Full Altitude Range	0.04	0.04	0.00	0.00	0.05	-0.08	0.07

Table S6. *R* coefficients between several OMPS-LP parameters (SZA, SSA, Reflectance, etc.) and the relative differences (between OMPS-LP-derived estimates and balloon-borne measurements of ozone) over the period SON 2012-2018. *R* coefficients higher than 0.2 and lower than -0.2 are in bold.

SYOWA - SON							
Altitude (km)	Time Difference	Distance	SZA	SSA	Tropopause	Surface Reflectance	Cloud Height
12.5	-0.04	0.02	0.14	0.16	-0.16	0.18	-0.03
13.5	-0.08	-0.16	-0.15	-0.15	-0.08	0.01	0.08
14.5	-0.10	-0.01	**-0.25**	**-0.25**	0.11	0.07	0.09
15.5	**-0.20**	0.05	-0.04	-0.04	-0.05	0.11	-0.13
16.5	0.09	0.04	-0.03	-0.04	-0.16	-0.01	-0.09
17.5	-0.18	0.06	-0.15	-0.16	-0.01	0.01	-0.07
18.5	-0.18	0.13	0.12	0.11	-0.12	0.01	0.04
19.5	0.05	**0.25**	0.07	0.07	0.01	0.02	-0.06
20.5	**0.22**	-0.01	0.14	0.16	**0.28**	**0.30**	0.00
21.5	**0.35**	0.12	0.05	0.05	-0.11	-0.07	0.07
22.5	**0.32**	0.02	-0.15	-0.12	0.01	-0.19	0.20
23.5	0.13	-0.05	**-0.38**	**-0.36**	0.13	-0.07	**0.38**
24.5	0.15	-0.07	**-0.43**	**-0.41**	0.12	0.04	**0.35**
25.5	0.19	-0.11	**-0.44**	**-0.41**	0.04	0.06	**0.35**
26.5	**0.28**	-0.05	**-0.35**	**-0.32**	0.11	0.18	**0.25**
27.5	**0.21**	0.09	-0.07	-0.07	0.17	0.14	0.04
Full Altitude Range	-0.03	0.02	-0.06	-0.06	-0.01	0.04	0.00

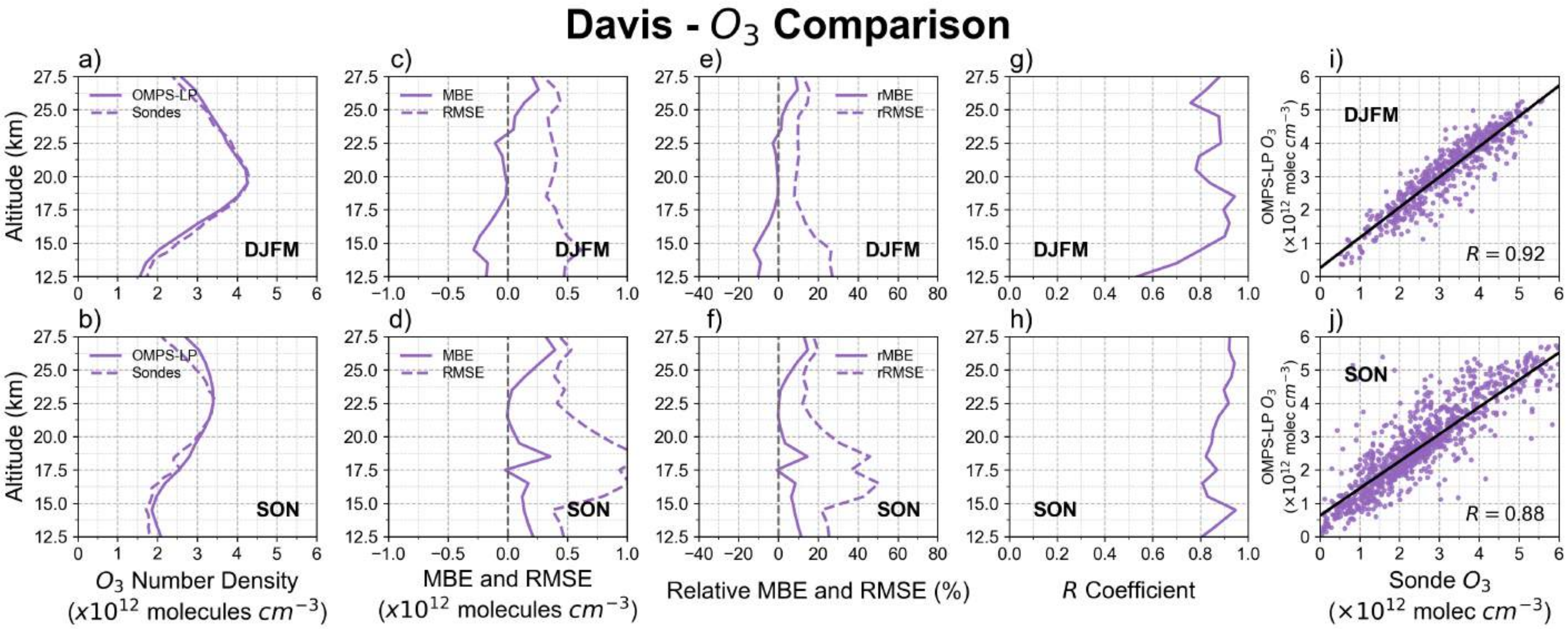

Fig. S11. Comparison between OMPS-LP-derived and balloon-borne data of ozone over the period DJFM 2013-2019 (first row), and over the period SON 2012-2018 (second row) for Davis Station.

a-b) Mean profiles. The solid line stands for the MEAN computed from OMPS-LP-derived estimates of ozone and the dashed line stands for the MEAN computed from balloon-borne measurements of ozone. 47 and 81 ozone profiles were compared over the periods DJFM 2013-2019 and SON 2012-2018, respectively;
c-d) Mean Bias Error (*MBE*, solid line) and Root Mean Square Error (*RMSE*, dashed line) relative to balloon-borne ozone data;
e-f) Relative Mean Bias Error (*MBE*, solid line) and Relative Root Mean Square Error (*RMSE*, dashed line);
g-h) *R* profile;
i-j) Scatter plot (the correlation coefficient is shown in the plot).

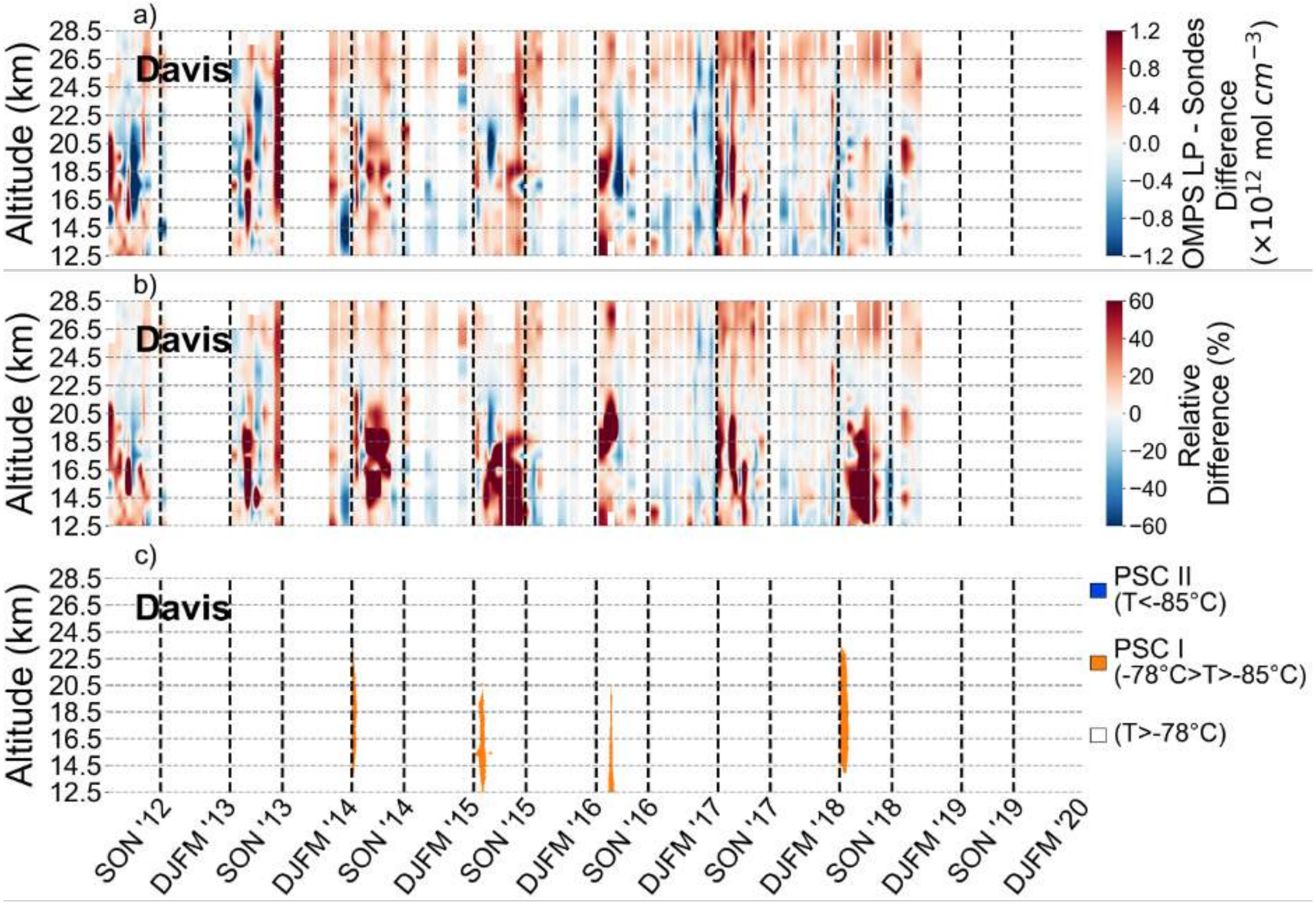

Fig. S12. Heatmap of the differences (absolute and relative) between OMPS-LP-derived estimates and balloon-borne measurements; blank spaces indicates periods within which no sondes fulfilling the comparison criteria were available. Periods and altitudes at which the temperature favors the formation of polar stratospheric clouds (PSC) are shown below the heatmaps; temperature profiles from sondes were used. Dashed lines separate different periods (DJFM and SON).

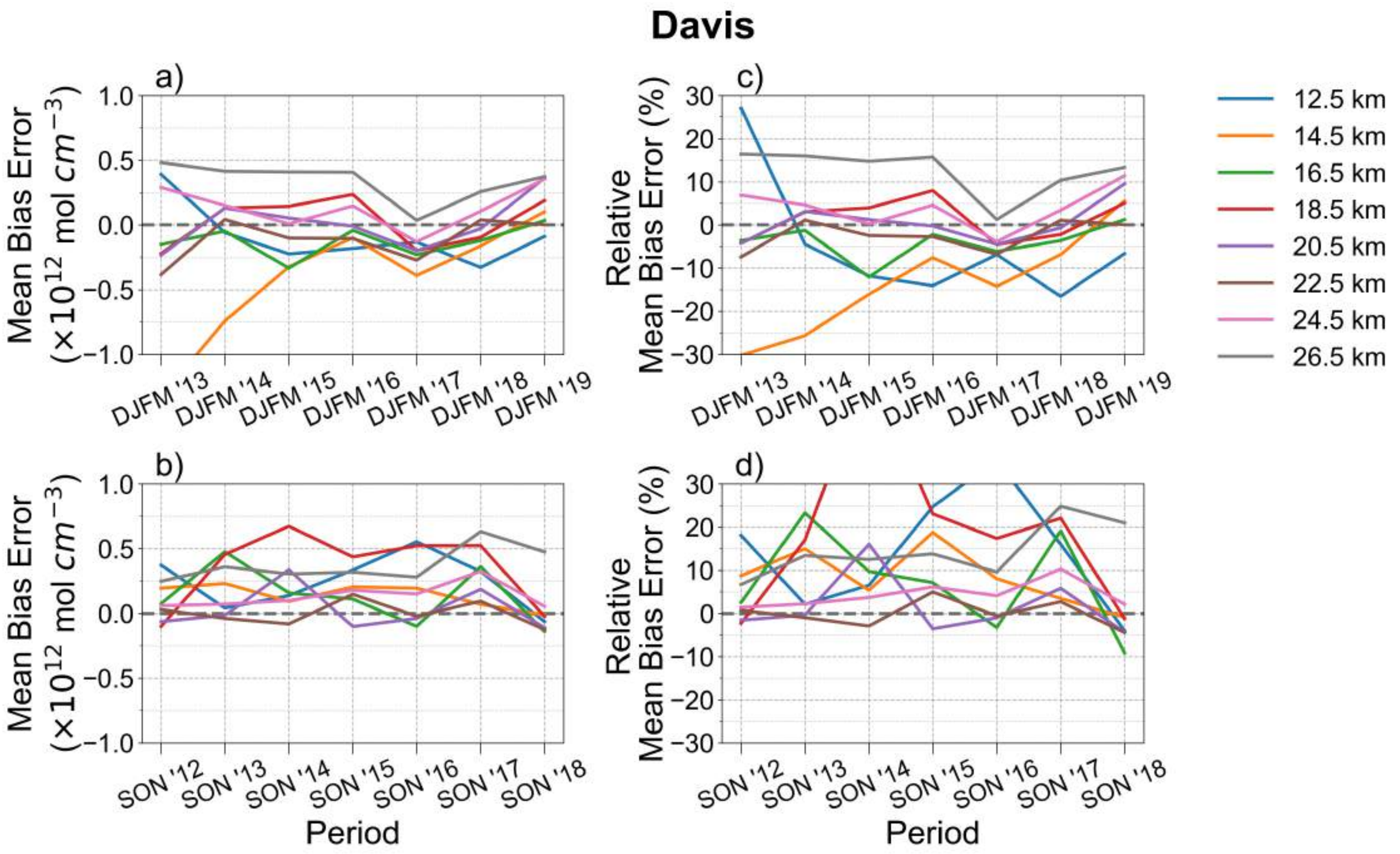

Fig. S13. Time series of the absolute and relative mean bias errors (*MBE*) of OMPS-LP-derived estimates of the ozone (relative to balloon-borne data) computed for Davis Station, over the DJFM period (first row) and over the SON period (second row).

Table S7. *R* coefficients between several OMPS-LP parameters (SZA, SSA, Reflectance, etc.) and the relative differences (between the OMPS-LP-derived estimates and the balloon-borne measurements of ozone) over the period DJFM 2013-2020. *R* coefficients higher than 0.2 and lower than -0.2 are in bold.

DAVIS - DJFM							
Altitude (km)	Time Difference	Distance	SZA	SSA	Tropopause	Surface Reflectance	Cloud Height
12.5	-0.10	-0.09	0.06	0.07	0.09	0.12	0.01
13.5	0.05	-0.10	-0.07	-0.08	0.17	0.12	-0.16
14.5	0.20	-0.18	**-0.23**	-0.16	-0.01	**0.26**	-0.03
15.5	**-0.28**	**-0.30**	**-0.25**	**-0.24**	0.20	**0.28**	0.09
16.5	0.04	0.08	-0.08	-0.04	0.11	**0.32**	0.00
17.5	-0.02	-0.16	0.09	0.15	-0.13	-0.08	0.06
18.5	**-0.43**	0.02	**-0.36**	**-0.42**	**0.33**	0.09	**-0.21**
19.5	-0.05	-0.06	**-0.25**	**-0.26**	0.10	-0.04	**-0.28**
20.5	-0.06	0.04	**-0.36**	**-0.37**	0.06	0.02	**-0.25**
21.5	**-0.22**	0.04	-0.02	-0.06	-0.07	0.03	**-0.28**
22.5	0.00	0.05	0.00	-0.01	-0.04	0.03	**-0.34**
23.5	0.10	-0.06	-0.08	-0.09	-0.03	0.03	**-0.33**
24.5	**-0.21**	**-0.27**	**-0.56**	**-0.55**	0.06	-0.12	-0.04
25.5	**-0.42**	-0.11	**-0.49**	**-0.50**	-0.15	-0.09	-0.09
26.5	**-0.39**	-0.17	**-0.44**	**-0.45**	**-0.21**	-0.07	-0.08
27.5	-0.12	-0.16	**-0.35**	**-0.30**	-0.17	0.00	0.03
Full Altitude Range	-0.09	-0.09	-0.17	-0.16	0.03	0.07	-0.09

Table S8. *R* coefficients between several OMPS-LP parameters (SZA, SSA, Reflectance, etc.) and the relative differences (between the OMPS-LP-derived estimates and the balloon-borne measurements of ozone) over the period SON 2012-2018. *R* coefficients higher than 0.2 and lower than -0.2 are in bold.

DAVIS - SON							
Altitude (km)	Time Difference	Distance	SZA	SSA	Tropopause	Surface Reflectance	Cloud Height
12.5	**-0.25**	-0.16	0	0.02	-0.08	-0.16	**-0.26**
13.5	-0.07	-0.11	-0.19	-0.18	-0.11	-0.03	**-0.21**
14.5	0.05	0.02	-0.07	-0.08	0.01	0.13	**-0.21**
15.5	-0.07	-0.17	-0.02	-0.04	-0.06	0.1	-0.16
16.5	0.02	-0.15	0.02	0.02	-0.11	0.09	-0.1
17.5	-0.06	-0.11	0.09	0.08	-0.16	**0.24**	**-0.21**
18.5	0	-0.14	0.1	0.08	-0.13	0.15	**-0.21**
19.5	0.15	-0.03	0.1	0.1	**0.21**	0.19	**0.28**
20.5	-0.05	0.02	0.1	0.09	-0.07	0.19	0.09
21.5	0.05	**-0.23**	-0.01	-0.01	-0.05	0.08	0.02
22.5	-0.02	-0.19	**-0.25**	**-0.23**	-0.14	-0.13	-0.02
23.5	-0.08	-0.18	**-0.3**	**-0.29**	-0.15	-0.05	-0.02
24.5	0.01	**-0.21**	**-0.3**	**-0.28**	**-0.28**	0.02	0.12
25.5	0.04	-0.11	**-0.27**	**-0.26**	-0.2	0.1	0.04
26.5	**0.26**	-0.12	-0.15	-0.14	**-0.24**	0.17	0.2
27.5	**0.31**	0.04	0.13	0.12	0.03	**0.32**	**0.23**
Full Altitude Range	0	-0.08	0	0	-0.04	0.09	-0.06

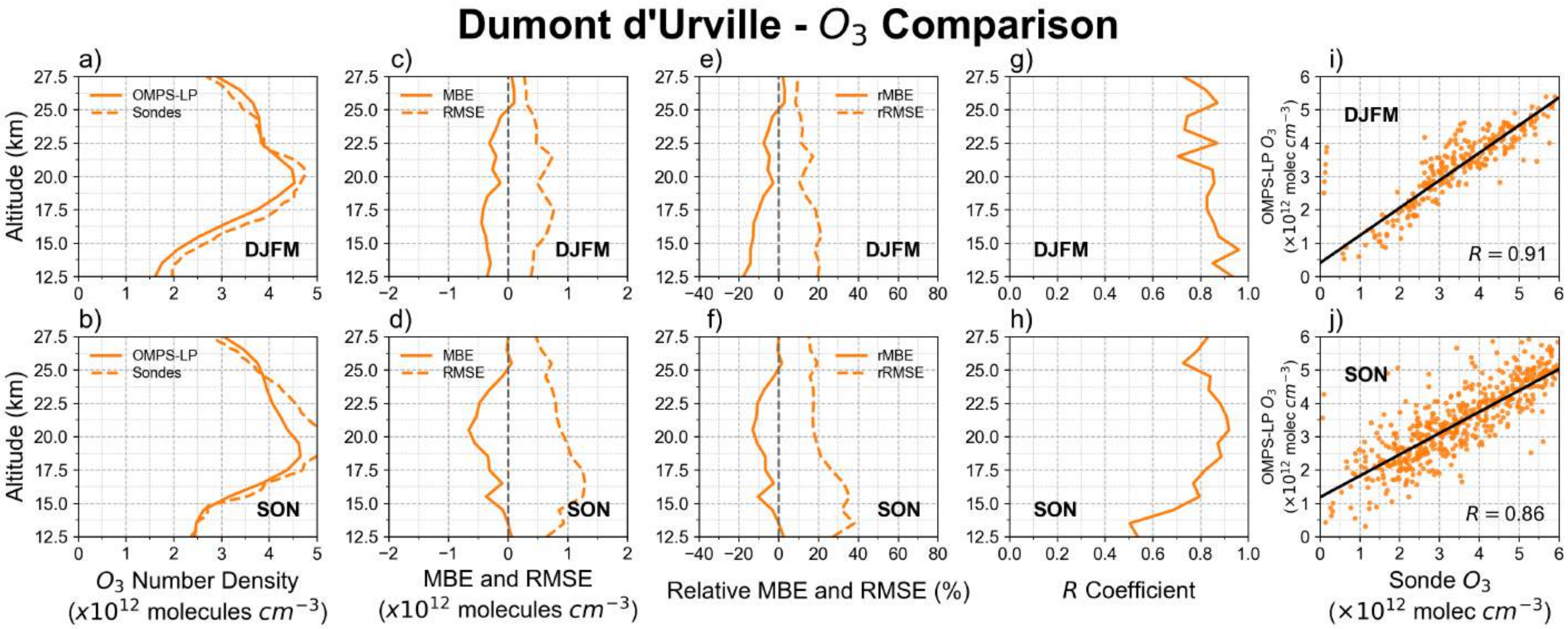

Fig. S14. Comparison between OMPS-LP-derived and balloon-borne data of ozone over the period DJFM 2013-2016 (first row) and over the period SON 2012-2018 (second row) for Dumont d'Urville Station.

a-b) Mean profiles. The solid line stands for the MEAN computed from OMPS-LP-derived estimates of ozone and the dashed line stands for the MEAN computed from balloon-borne measurements of ozone. 16 and 37 ozone profiles were compared over the periods DJFM 2013-2016 and SON 2012-2018, respectively;
c-d) Mean Bias Error (*MBE*, solid line) and Root Mean Square Error (*RMSE*, dashed line) relative to balloon-borne ozone data;
e-f) Relative Mean Bias Error (*MBE*, solid line) and Relative Root Mean Square Error (*RMSE*, dashed line);
g-h) *R* profile;
i-j) Scatter plot (the correlation coefficient is shown in the plot).

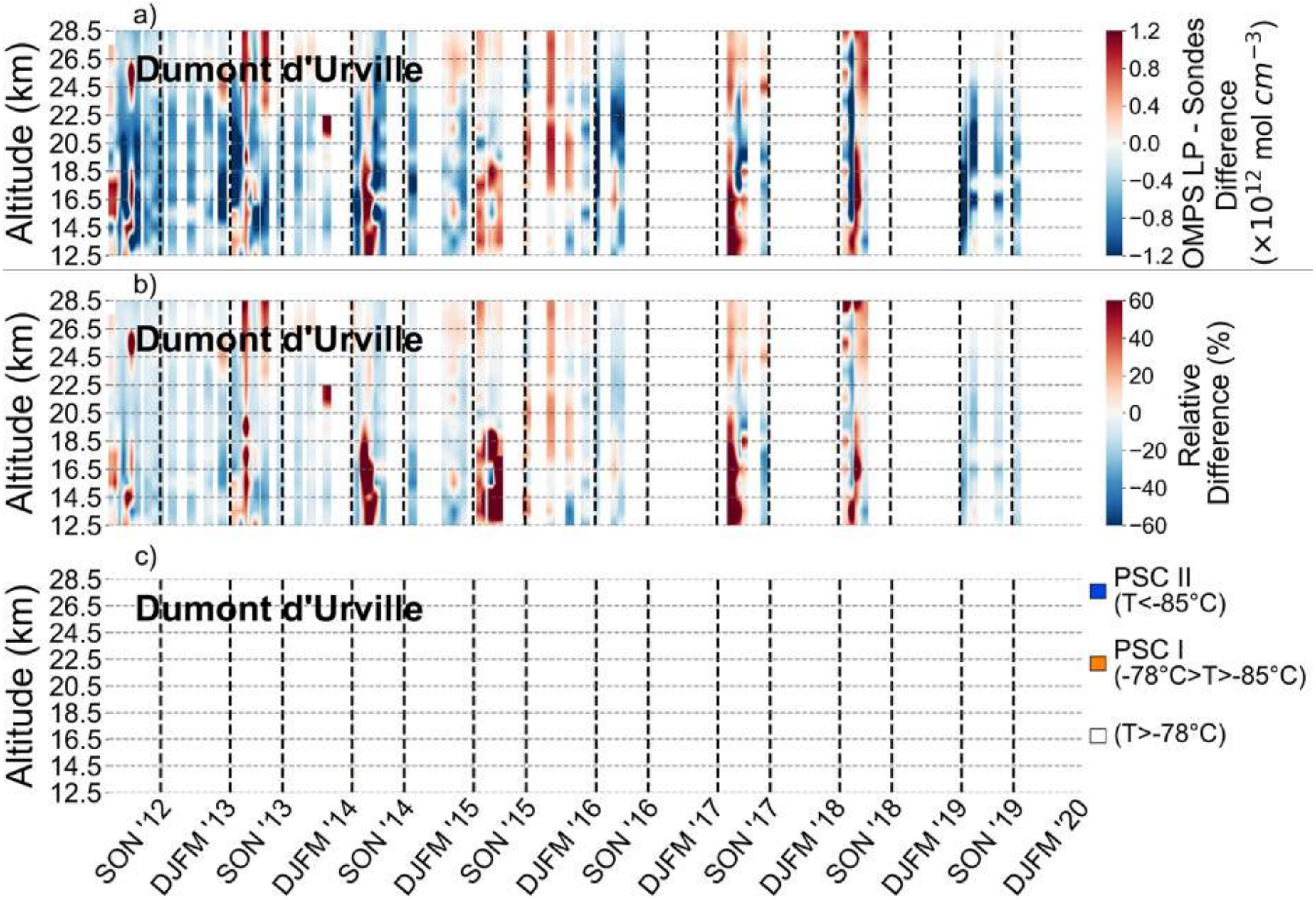

Fig. S15. Heatmap of the differences (absolute and relative) between OMPS-LP-derived estimates and balloon-borne measurements; blank spaces indicates periods within which no sondes fulfilling the comparison criteria were available. Periods and altitudes at which the temperature favors the formation of polar stratospheric clouds (PSC) are shown below the heatmaps; temperature profiles from sondes were used. Dashed lines separate different periods (DJFM and SON).

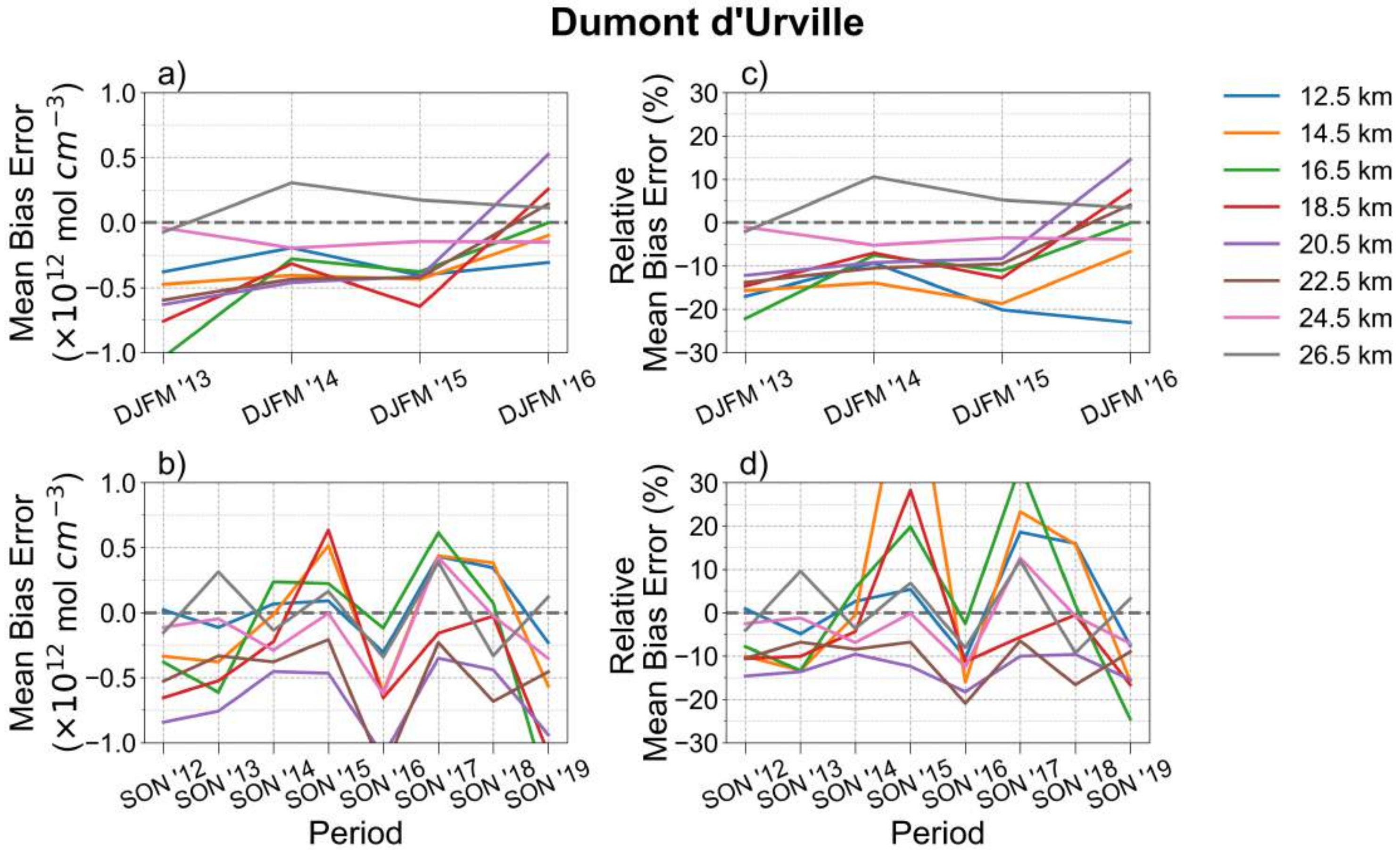

Fig. S16. Time series of the absolute and relative mean bias errors (*MBE*) of OMPS-LP-derived estimates of the ozone (relative to balloon-borne data) computed for Dumont d'Urville Station, over the DJFM period (first row) and over the SON period (second row).

Table S9. *R* coefficients between several OMPS-LP parameters (SZA, SSA, Reflectance, etc.) and the relative differences (between the OMPS-LP-derived estimates and the balloon-borne measurements of ozone) over the period DJFM 2013-2020. *R* coefficients higher than 0.2 and lower than -0.2 are in bold.

DUMONT D'URVILLE - DJFM							
Altitude (km)	Time Difference	Distance	SZA	SSA	Tropopause	Surface Reflectance	Cloud Height
12.5	**-0.24**	0.00	-0.13	-0.10	**-0.47**	0.19	-0.03
13.5	0.14	-0.12	-0.01	-0.07	**-0.30**	0.16	**-0.39**
14.5	**0.39**	0.00	-0.07	-0.15	-0.06	-0.15	**-0.39**
15.5	0.09	**0.29**	-0.15	-0.05	0.08	**-0.53**	-0.09
16.5	0.01	0.06	0.04	0.10	0.01	**-0.44**	-0.03
17.5	0.05	**-0.25**	-0.17	-0.11	0.19	0.06	0.03
18.5	-0.14	-0.20	**-0.25**	**-0.21**	0.17	0.05	-0.03
19.5	0.11	-0.14	**-0.21**	-0.19	**0.35**	-0.19	-0.07
20.5	**0.22**	-0.20	**-0.22**	**-0.21**	**0.36**	0.02	-0.09
21.5	0.16	0.06	-0.17	-0.13	0.10	-0.12	0.02
22.5	**0.29**	-0.10	-0.15	-0.16	**0.34**	-0.19	**-0.31**
23.5	**0.46**	**0.55**	0.01	-0.01	-0.06	**-0.31**	**-0.53**
24.5	0.06	**0.58**	0.19	**0.24**	**-0.33**	-0.07	**-0.32**
25.5	**-0.23**	**0.46**	**0.38**	**0.41**	**-0.41**	-0.02	**-0.31**
26.5	**-0.36**	**0.28**	0.03	0.10	**-0.23**	-0.06	-0.02
27.5	**-0.20**	0.19	-0.11	-0.03	-0.08	-0.15	-0.20
Full Altitude Range	0.07	0.06	-0.07	-0.05	0.00	-0.10	-0.15

Table S10. *R* coefficients between several OMPS-LP parameters (SZA, SSA, Reflectance, etc.) and the relative differences (between the OMPS-LP-derived estimates and the balloon-borne measurements of ozone) over the period SON 2012-2018. *R* coefficients higher than 0.2 and lower than -0.2 are in bold.

DUMONT D'URVILLE - SON							
Altitude (km)	Time Difference	Distance	SZA	SSA	Tropopause	Surface Reflectance	Cloud Height
12.5	-0.06	0.12	**0.23**	**0.22**	-0.12	**0.28**	**-0.20**
13.5	**0.27**	0.18	**0.24**	0.20	0.04	**0.45**	-0.16
14.5	0.18	**0.22**	0.16	0.12	-0.02	**0.47**	-0.13
15.5	0.13	0.03	**0.22**	**0.21**	-0.11	0.13	-0.03
16.5	0.10	0.14	0.13	0.10	-0.12	0.18	-0.06
17.5	0.09	0.18	**0.22**	**0.20**	-0.04	0.16	-0.01
18.5	**0.26**	0.13	0.10	0.07	0.11	**0.34**	-0.07
19.5	-0.03	0.09	0.09	0.09	-0.09	0.15	0.01
20.5	-0.08	0.05	0.02	0.00	0.02	0.12	0.06
21.5	0.04	**-0.28**	0.02	0.01	0.11	**0.21**	-0.07
22.5	-0.09	**-0.33**	-0.08	-0.09	0.00	0.10	0.06
23.5	-0.07	**-0.27**	-0.15	-0.15	-0.08	0.11	0.08
24.5	-0.04	-0.19	-0.16	-0.15	0.08	0.08	0.13
25.5	0.11	0.05	0.06	0.07	**0.26**	0.10	-0.04
26.5	-0.13	**-0.32**	-0.02	-0.01	0.01	0.15	0.07
27.5	0.02	**-0.27**	0.13	0.13	**0.20**	**0.31**	0.04
Full Altitude Range	0.08	0.06	0.11	0.09	-0.01	**0.20**	-0.04

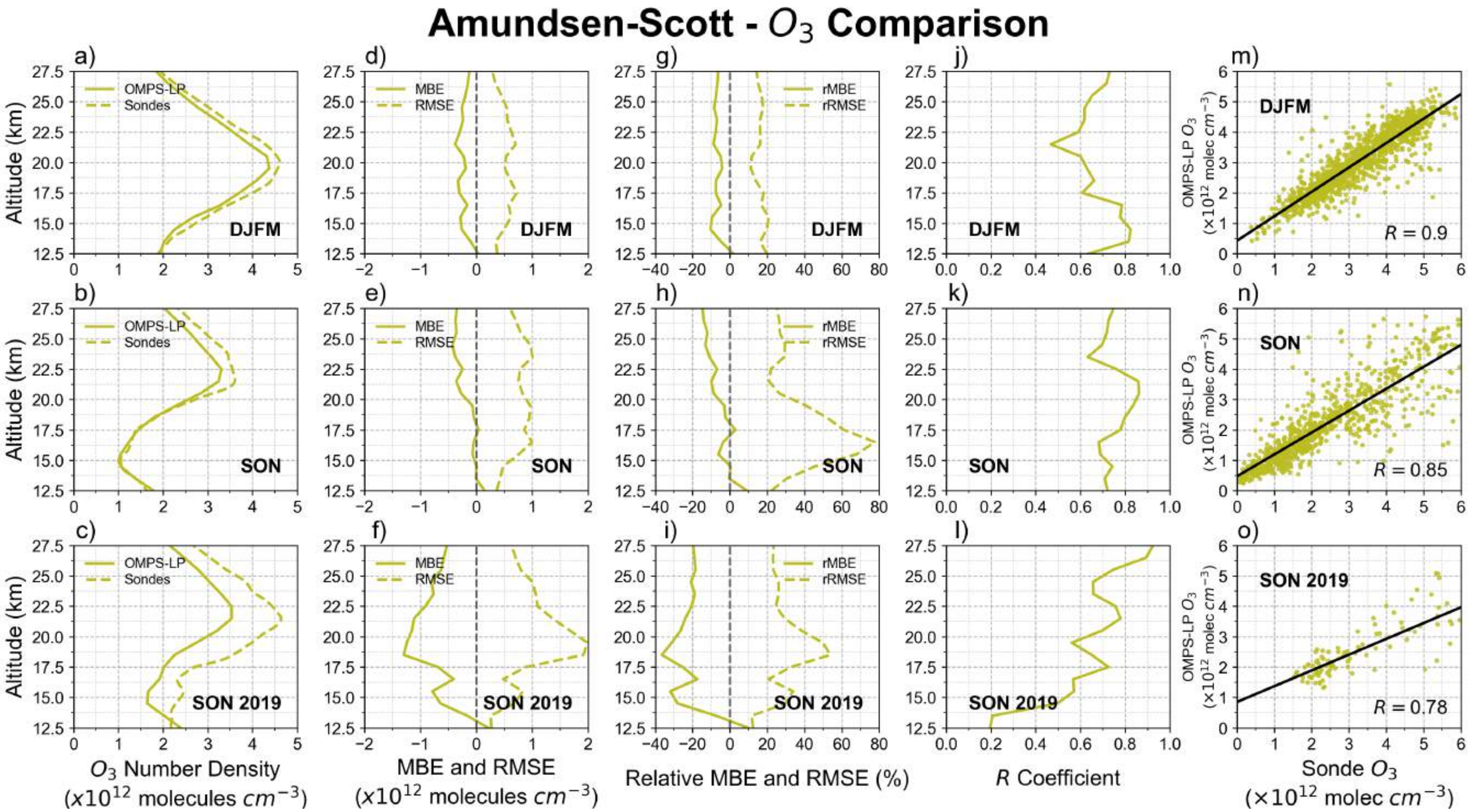

Fig. S17. Comparison between OMPS-LP-derived and balloon-borne data of ozone over the period DJFM 2013-2020 (first row), over the period SON 2012-2018 (second row) and over the period SON 2019 (third row) for Amundsen-Scott Station.

a-c) Mean profiles. The solid line stands for the MEAN computed from OMPS-LP-derived data of ozone and the dashed line stands for the MEAN computed from balloon-borne data of ozone. 74, 49 and 6 ozone profiles were compared over the periods DJFM 2013-2020, SON 2012-2018 and SON 2019, respectively;

d-f) Mean Bias Error (*MBE*, solid line) and Root Mean Square Error (*RMSE*, dashed line) relative to balloon-borne ozone data;

g-i) Relative Mean Bias Error (*MBE*, solid line) and Relative Root Mean Square Error (*RMSE*, dashed line);

j-l) *R* profile;

m-o) Scatter plot (the correlation coefficient is shown in the plot).

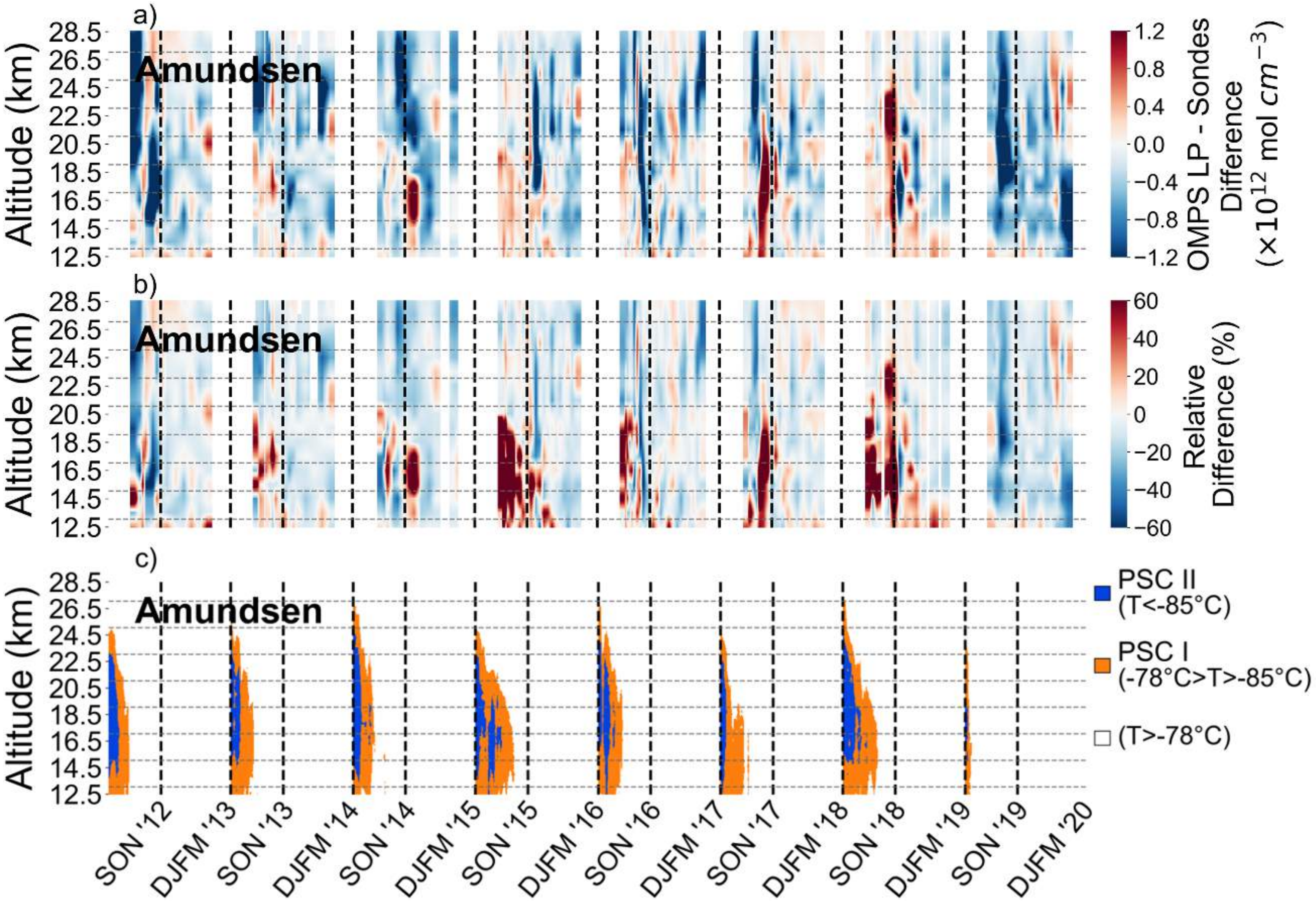

Fig. S18. Heatmap of the differences (absolute and relative) between OMPS-LP-derived estimates and balloon-borne measurements; blank spaces indicates periods within which no sondes fulfilling the comparison criteria were available. Periods and altitudes at which the temperature favors the formation of polar stratospheric clouds (PSC) are shown below the heatmaps; temperature profiles from sondes were used. Dashed lines separate different periods (DJFM and SON).

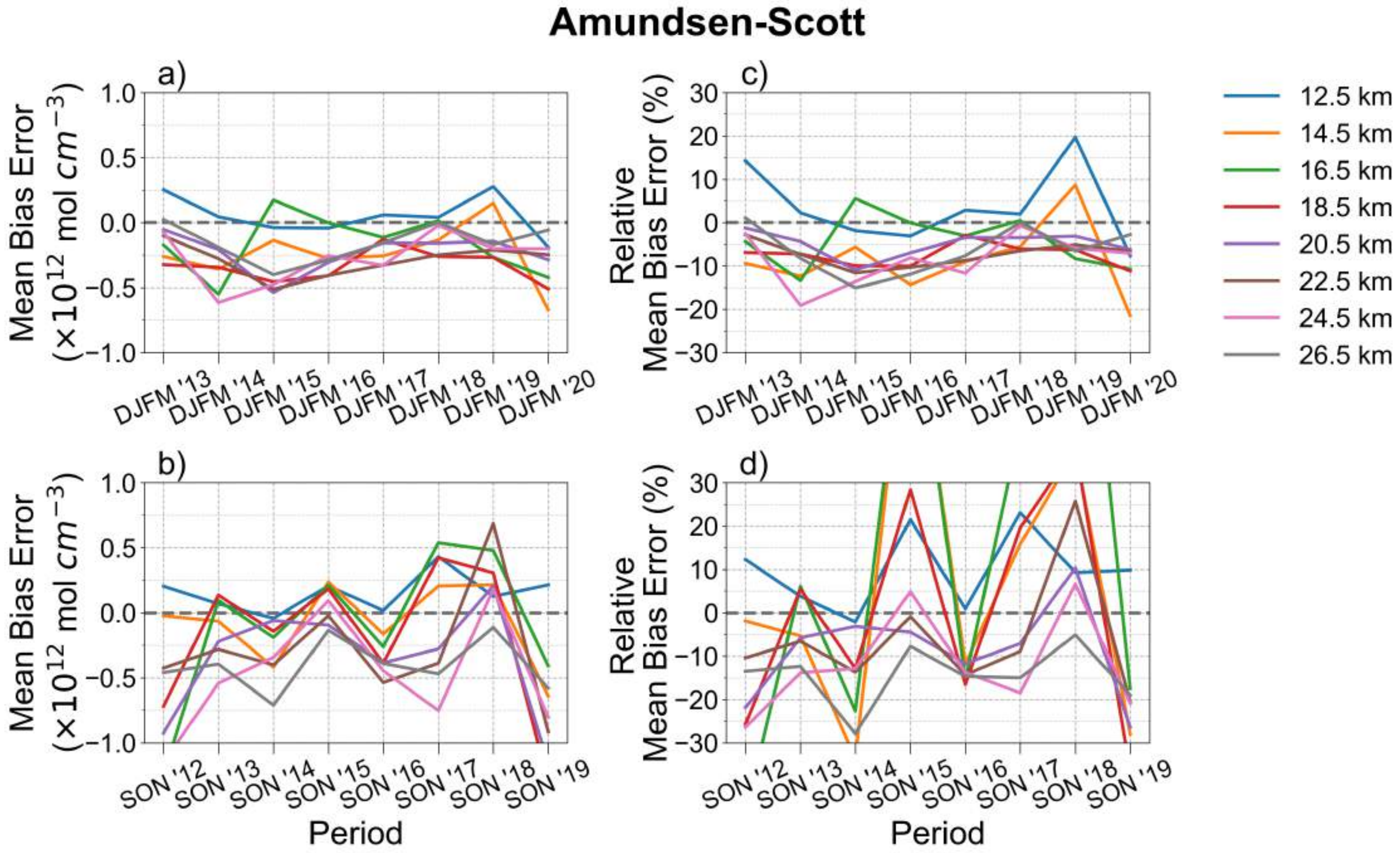

Fig. S19. Time series of the absolute and relative mean bias errors (*MBE*) of OMPS-LP-derived estimates of the ozone (relative to balloon-borne data) computed for Amundsen-Scott Station, over the DJFM period (first row) and over the SON period (second row).

Table S11. *R* coefficients between several OMPS-LP parameters (SZA, SSA, Reflectance, etc.) and the relative differences (between the OMPS-LP-derived estimates and the balloon-borne measurements of ozone) over the period DJFM 2013-2020. *R* coefficients higher than 0.2 and lower than -0.2 are in bold.

AMUNDSEN-SCOTT - DJFM							
Altitude (km)	Time Difference	Distance	SZA	SSA	Tropopause	Surface Reflectance	Cloud Height
12.5	-0.14	0.07	-0.15	-0.13	0.03	**0.21**	0.01
13.5	-0.10	-0.03	-0.15	-0.16	0.04	0.17	-0.07
14.5	-0.11	-0.13	**-0.24**	**-0.29**	**0.27**	**0.24**	-0.02
15.5	-0.10	**-0.26**	**-0.25**	**-0.31**	**0.25**	0.10	0.07
16.5	-0.10	**-0.35**	**-0.26**	**-0.28**	0.16	0.12	0.04
17.5	-0.04	**-0.21**	-0.13	-0.16	-0.02	-0.02	0.12
18.5	-0.20	0.00	-0.03	-0.03	**0.22**	0.07	0.01
19.5	**-0.20**	0.03	0.00	-0.04	**0.30**	0.04	0.10
20.5	-0.10	0.15	**0.32**	**0.33**	0.07	-0.05	0.00
21.5	-0.10	0.15	0.15	0.15	0.13	0.05	0.00
22.5	0.07	0.11	0.04	0.02	0.15	0.03	-0.04
23.5	**0.23**	-0.10	-0.04	-0.05	0.15	-0.01	-0.10
24.5	0.06	-0.12	-0.20	-0.18	0.18	0.18	0.06
25.5	-0.19	-0.08	**-0.24**	-0.17	0.07	**0.39**	0.05
26.5	**-0.27**	0.09	**-0.26**	-0.18	0.02	**0.50**	0.13
27.5	**-0.31**	**0.23**	**-0.37**	**-0.26**	0.08	**0.62**	0.10
Full Altitude Range	-0.09	-0.06	-0.13	-0.13	0.12	0.15	0.03

Table S12. *R* coefficients between several OMPS-LP parameters (SZA, SSA, Reflectance, etc.) and the relative differences (between the OMPS-LP-derived estimates and the balloon-borne measurements of ozone) over the period SON 2012-2018. *R* coefficients higher than 0.2 and lower than -0.2 are in bold.

AMUNDSEN-SCOTT - SON							
Altitude (km)	Time Difference	Distance	SZA	SSA	Tropopause	Surface Reflectance	Cloud Height
12.5	**-0.22**	-0.13	0.19	**0.22**	**0.26**	**0.25**	0.08
13.5	0.08	-0.2	0.17	0.16	**0.37**	0.17	-0.04
14.5	0.03	**-0.22**	**0.25**	**0.26**	**0.49**	0.07	0.04
15.5	-0.02	**-0.32**	**0.22**	0.19	**0.61**	0.13	-0.05
16.5	0.05	**-0.24**	**0.25**	**0.22**	**0.57**	-0.11	-0.07
17.5	-0.16	-0.12	0.17	0.16	**0.46**	-0.06	-0.13
18.5	-0.09	-0.17	**0.26**	**0.23**	**0.41**	0.01	-0.13
19.5	-0.06	**-0.23**	**0.41**	**0.37**	**0.56**	-0.14	-0.17
20.5	**-0.22**	-0.08	0.18	0.12	0.11	-0.07	-0.08
21.5	-0.09	0.04	**-0.24**	-0.19	0.07	0.13	-0.06
22.5	-0.18	0.05	**-0.34**	**-0.3**	-0.06	**0.33**	-0.06
23.5	-0.15	0	**-0.25**	**-0.27**	0.09	**0.32**	-0.09
24.5	-0.04	-0.04	**-0.25**	**-0.3**	0.12	**0.3**	-0.18
25.5	-0.03	0.04	**-0.33**	**-0.31**	-0.03	**0.29**	**-0.32**
26.5	-0.02	0.2	**-0.33**	**-0.27**	-0.09	0.19	**-0.45**
27.5	-0.04	**0.23**	**-0.38**	**-0.32**	-0.07	0.14	**-0.45**
Full Altitude Range	-0.03	-0.13	0.11	0.1	**0.31**	0.04	-0.07

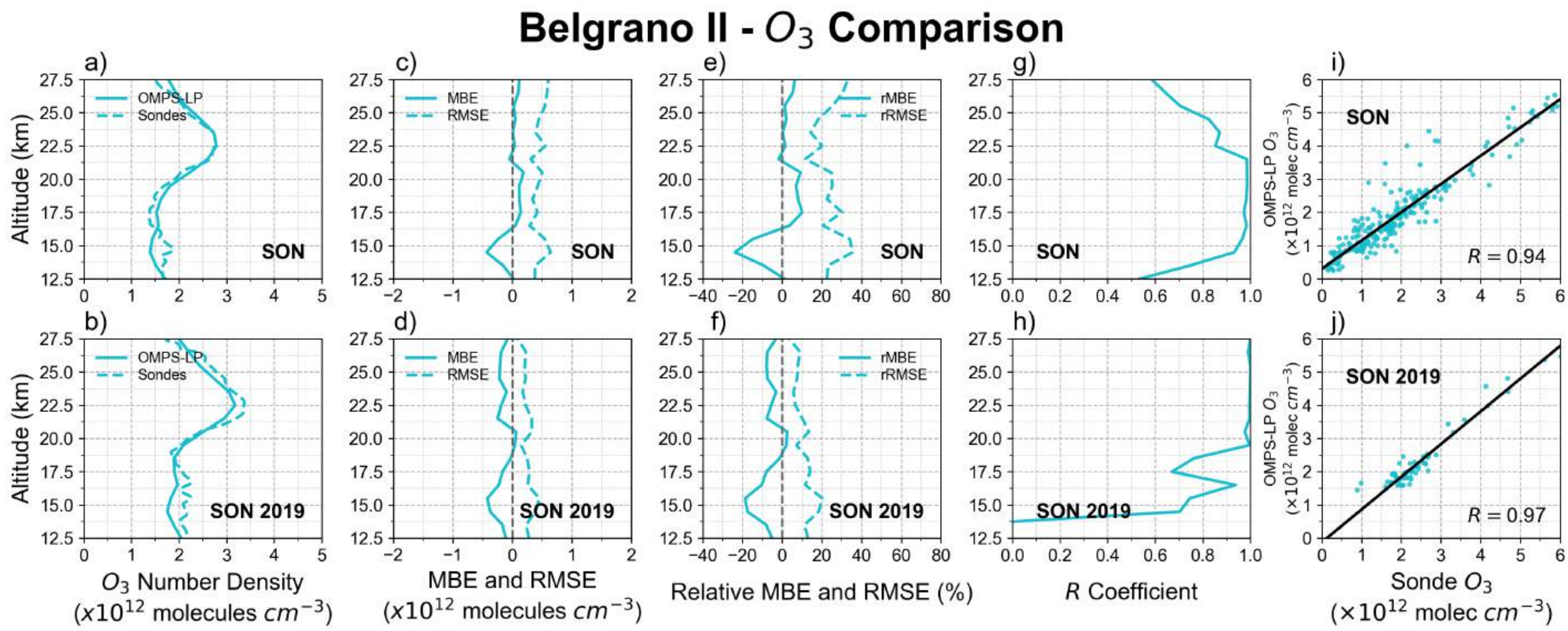

Fig. S20. Comparison between OMPS-LP-derived and balloon-borne data of ozone over the period SON 2016-2018 (first row) and over the period SON 2019 (second row) for Belgrano II Station.

a-b) Mean profiles. The solid line stands for the MEAN computed from OMPS-LP-derived data of ozone and the dashed line stands for the MEAN computed from balloon-borne data of ozone. 15 and 4 ozone profiles were compared over the periods SON 2016-2018 and SON 2019, respectively;
c-d) Mean Bias Error (*MBE*, solid line) and Root Mean Square Error (*RMSE*, dashed line) relative to balloon-borne ozone data;
e-f) Relative Mean Bias Error (*MBE*, solid line) and Relative Root Mean Square Error (*RMSE*, dashed line);
g-h) *R* profile;
i-j) Scatter plot (the correlation coefficient *R* is shown in the plot).

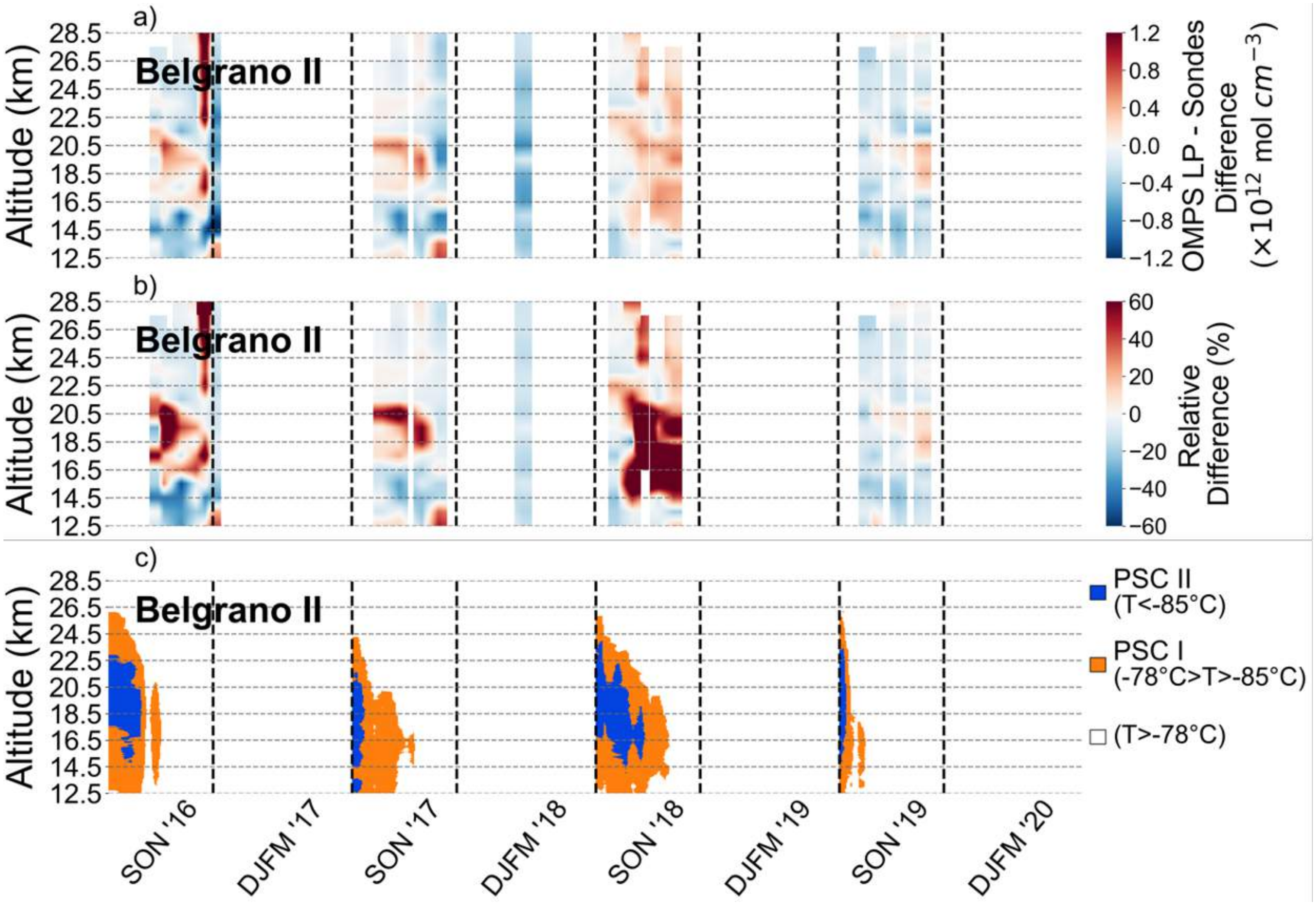

Fig. S21. Heatmap of the differences (absolute and relative) between OMPS-LP-derived estimates and balloon-borne measurements; blank spaces indicates periods within which no sondes fulfilling the comparison criteria were available. Periods and altitudes at which the temperature favors the formation of polar stratospheric clouds (PSC) are shown below the heatmaps; temperature profiles from sondes were used. Dashed lines separate different periods (DJFM and SON).

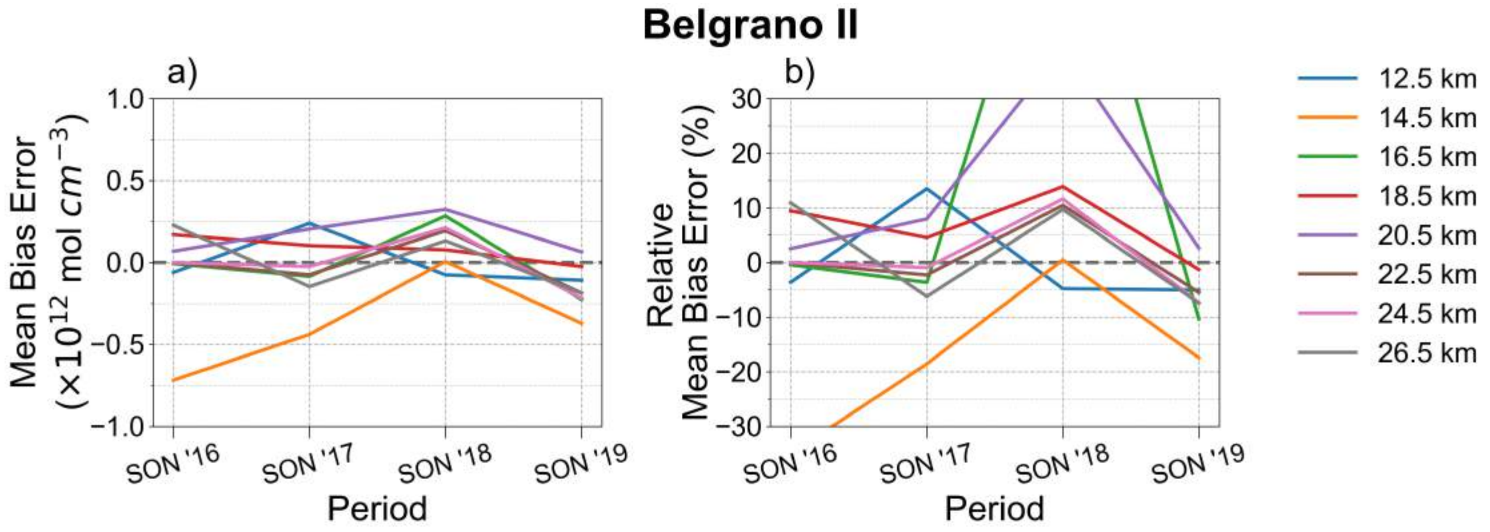

Fig. S22. Time series of the absolute and relative mean bias errors (*MBE*) of OMPS-LP-derived estimates of the ozone (relative to balloon-borne data) computed for Belgrano II Station, over the SON period.

Table S13. *R* coefficients between several OMPS-LP parameters (SZA, SSA, Reflectance, etc.) and the relative differences (between the OMPS-LP-derived estimates and the balloon-borne measurements of ozone) over the period SON 2012-2018. *R* coefficients higher than 0.2 and lower than -0.2 are in bold.

BELGRANO II - SON							
Altitude (km)	Time Difference	Distance	SZA	SSA	Tropopause	Surface Reflectance	Cloud Height
12.5	**0.34**	-0.2	0.15	0.05	**-0.39**	**-0.44**	0.01
13.5	-0.14	-0.2	0.04	0.01	-0.17	**-0.46**	-0.14
14.5	0	0.04	**0.34**	**0.27**	**0.54**	**0.28**	**0.24**
15.5	**-0.24**	-0.16	**0.27**	**0.23**	**0.78**	**0.24**	-0.17
16.5	**-0.49**	-0.03	-0.02	-0.1	**0.68**	0.17	-0.12
17.5	-0.16	**-0.21**	0.1	0.01	**0.49**	0.2	0.04
18.5	-0.02	-0.02	-0.05	-0.03	0.11	0.16	**0.56**
19.5	0.01	-0.06	0.02	-0.02	**0.29**	**0.41**	**0.50**
20.5	**-0.23**	-0.15	0.16	**0.24**	**0.57**	**0.55**	**0.42**
21.5	-0.08	-0.18	**0.45**	**0.5**	**0.56**	**0.34**	0.13
22.5	**0.48**	0.17	**0.55**	**0.54**	-0.01	-0.05	0
23.5	**0.64**	**-0.3**	**0.34**	**0.24**	-0.12	0.09	-0.13
24.5	**0.3**	-0.04	**0.29**	**0.27**	0.12	0.12	**0.52**
25.5	**0.51**	-0.01	**0.25**	**0.22**	-0.07	0	**0.21**
26.5	**0.49**	-0.02	**0.22**	**0.22**	-0.05	0.01	**0.24**
27.5	**0.68**	-0.17	**0.22**	0.19	-0.09	0	-0.1
Full Altitude Range	-0.01	-0.08	0.13	0.1	**0.27**	0.13	0.17

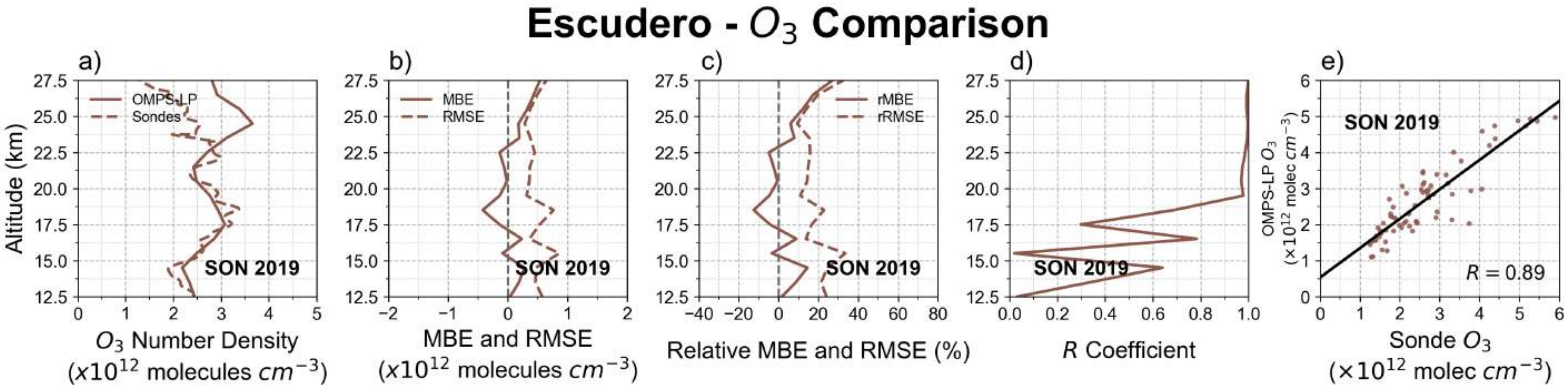

Fig. S23. Comparison between OMPS-LP-derived and balloon-borne data of ozone over the period SON 2019 for Escudero Station.
a) Mean profiles. The solid line stands for the MEAN computed from OMPS-LP-derived data of ozone and the dashed line stands for the MEAN computed from balloon-borne data of ozone. 5 ozone profiles were compared over the period SON 2019;
b) Mean Bias Error (*MBE*, solid line) and Root Mean Square Error (*RMSE*, dashed line) relative to balloon-borne ozone data;
c) Relative Mean Bias Error (*MBE*, solid line) and Relative Root Mean Square Error (*RMSE*, dashed line);
d) *R* profile;
e) Scatter plot (the correlation coefficient *R* is shown in the plot).
